# Scaling the Process Chemistry of a COVID-19 Antiviral Pharmaceutical Down for a Multistep Synthesis Experiment in the Undergraduate Teaching Laboratory

**DOI:** 10.1021/acs.jchemed.3c00999

**Published:** 2024-02-07

**Authors:** Andrew J. Wommack, Aaliyah B. Holloway, Kaitlyn A. Stallings, Pamela M. Lundin

**Affiliations:** † Department of Chemistry, High Point University, High Point, North Carolina 27268, United States; ‡ Cambrex, High Point, North Carolina 27265, United States

**Keywords:** Second-Year Undergraduate, Upper-Division Undergraduate, Organic Chemistry, Laboratory Instruction, Hands-On Learning/Manipulatives, Medicinal Chemistry

## Abstract

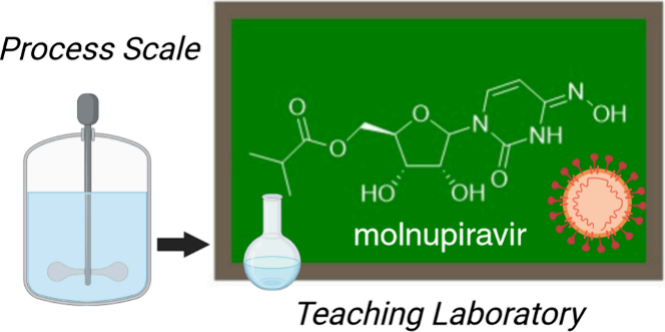

Molnupiravir is an orally bioavailable direct acting antiviral agent that received emergency use authorization in late 2021 from the FDA for the treatment of patients with mild, moderate, or severe COVID-19. This prodrug is metabolized into a ribonucleoside that is incorporated into the viral RNA during replication. Its tautomerization between cytidine- and uridine-like forms ultimately causes multiple irreversible errors in the genetic code of the virus, which prevents successful viral replication. There are multiple process chemistry routes for molnupiravir synthesis published in the literature that attempt to maximize synthetic yield while minimizing cost and waste, which are goals similar to those of an implementable educational laboratory experiment for the teaching laboratory. We have developed a multiweek laboratory module for undergraduate students in which students conduct a multistep synthesis of molnupiravir. Specifically, our Organic Chemistry II Laboratory students performed the final two steps of molnupiravir synthesis using procedures derived directly from the published process chemistry literature. We utilized this opportunity to introduce students to reading and interpreting these primary experimental sources. Students obtained authentic characterization data via high pressure liquid chromatography (HPLC) and nuclear magnetic resonance (NMR) spectroscopy to assess the conversion and purity of their products at each synthetic step. We report our in-lab activities and student generated data as well as suggestions for how this laboratory experiment could be tailored to meet similar learning objectives in other courses, such as medicinal chemistry or capstone laboratory courses, and as a function of available instrumentation.

## Introduction

As scientists, the COVID-19 pandemic has been an excellent opportunity to witness the scientific community coming together to solve global challenges, including the expeditious development of treatments, including vaccines and oral therapeutics. In the context of organic chemistry, the development of antiviral therapeutic agents that can be administered orally has been exciting to watch, especially given that the 21-month gap from the onset of the COVID-19 pandemic in the United States to the Emergency Use Authorizations for paxlovid^1^ and molnupiravir^2^ was significantly reduced as compared to the canonical timeline for developing a novel pharmaceutical treatment.

**Figure 1:**
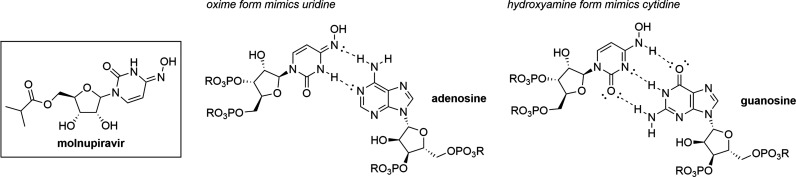
Molnupiravir structure and tautomerization to mimic U and C and form a stable base with A and G.

Molnupiravir, also known in the literature as EIDD-2801, is an esterified form of RNA nucleoside analog β-d-*N4*-hydroxycytidine. In vivo, it is rapidly converted into the triphosphate form, whose oxime tautomerization enables it to mimic both U and C and form stable base pairs with both A and G (Figure 1). The stability of these base pairs allow the mutations to escape viral proofreading and mutations quickly proliferate to shut down viral replication.^3^

In reading the literature for the process scale synthesis of molnupiravir,^4−7^ we were struck by how the ideal conditions for process scale chemistry mirrors those of the teaching lab: it is imperative that large quantities of materials must be procured at minimal cost, synthetic steps should proceed in high yield, and waste streams should be minimized. Additionally, many of the synthetic steps of molnupiravir synthesis are relevant to the standard organic chemistry curriculum, including functional group protection, esterification, nucleophilic displacement, and acid-mediated hydrolysis. The mechanism of action in which molnupiravir tautomerizes to mimic the base pairing of both U and C is also relevant to the organic chemistry curriculum.

In this laboratory experiment, we capitalize on these common interests and themes between process chemistry and the organic chemistry curriculum to develop a multistep synthesis experiment for organic chemistry laboratory students. The pandemic has been a cauldron of opportunity to bring cutting-edge science into the classroom and teaching laboratory, and the college student population over the next decade will be people whose education and personal lives have been highly impacted by the COVID-19 pandemic.^8^ Incorporating primary literature on the SARS-CoV-2 pandemic has been one way for students to engage with typical course material in ways that are relevant to them.^9−11^ The relative risks and benefits of prescribing molnupiravir to various populations continues to be explored by clinicians and scientists alike in the literature,^12−15^ giving students an opportunity to think more broadly about ethical considerations in developing treatments plans for patients with COVID-19. Utilizing SARS-CoV-2 literature in course material can have the added benefit of promoting and practicing science literacy,^9^ an issue that has been at the forefront of societal discussions as we have rapidly adapted our public health practices in response to the pandemic as well as grappled with other science-related issues with widespread impacts such as climate change.^16,17^

## Pedagogical Significance

This experiment was performed by students enrolled in a second semester organic chemistry laboratory course intended for biochemistry and chemistry majors at High Point University in the springs of 2022 and 2023. This one-credit course is taught by an instructor that meets for one 4 h session a week. The students performed the described activity over a four week period roughly halfway through the semester. In the prerequisite first semester organic chemistry laboratory course the previous semester, the students had learned basic synthetic chemistry techniques such as reaction setup, liquid–liquid extraction, and thin-layer chromatography. Prior to executing the described experiment in the second semester laboratory course, students had learned to operate the nuclear magnetic resonance (NMR) spectrometer and process the obtained data. In the corequisite Organic Chemistry II lecture course, students were concurrently learning nucleophilic condensation, nucleophilic acyl substitution, and acid-catalyzed hydrolysis, which are mechanisms that occur in the reactions the students performed. During the semester in which they performed this laboratory experiment, many of the students were also enrolled in a scientific research and writing course that is a requirement for the completion of our chemistry and biochemistry majors.

This experiment was designed to introduce students to the experience of multistep synthesis, which they had not previously encountered outside any research experience they might have had. Specifically, the students were asked to assess purity at each stage via high pressure liquid chromatography (HPLC) and NMR spectroscopy and calculate the overall percent yield. Doing this in two subsequent steps motivated the students to think about how impurities at one stage of a synthesis can be carried through subsequent steps and complicate downstream purification, emphasizing the importance of precise and thorough compound characterization at each synthetic step. As process-scale primary literature was used to craft the experimental procedure followed by the students, the students were asked to read selected passages from the Supporting Information and contrast the techniques and language to those they typically see in a laboratory handout, which dovetailed nicely with the content they were learning in their research and writing course. Finally, as many of these students enrolled in this course are interested in pursuing careers in the health professions, we also utilized this experiment as an opportunity to make interdisciplinary connections such as the biology of RNA virus replication and ethical questions regarding the approval and administration of pharmaceutical agents.

## Experimental Overview

**Scheme 1:**
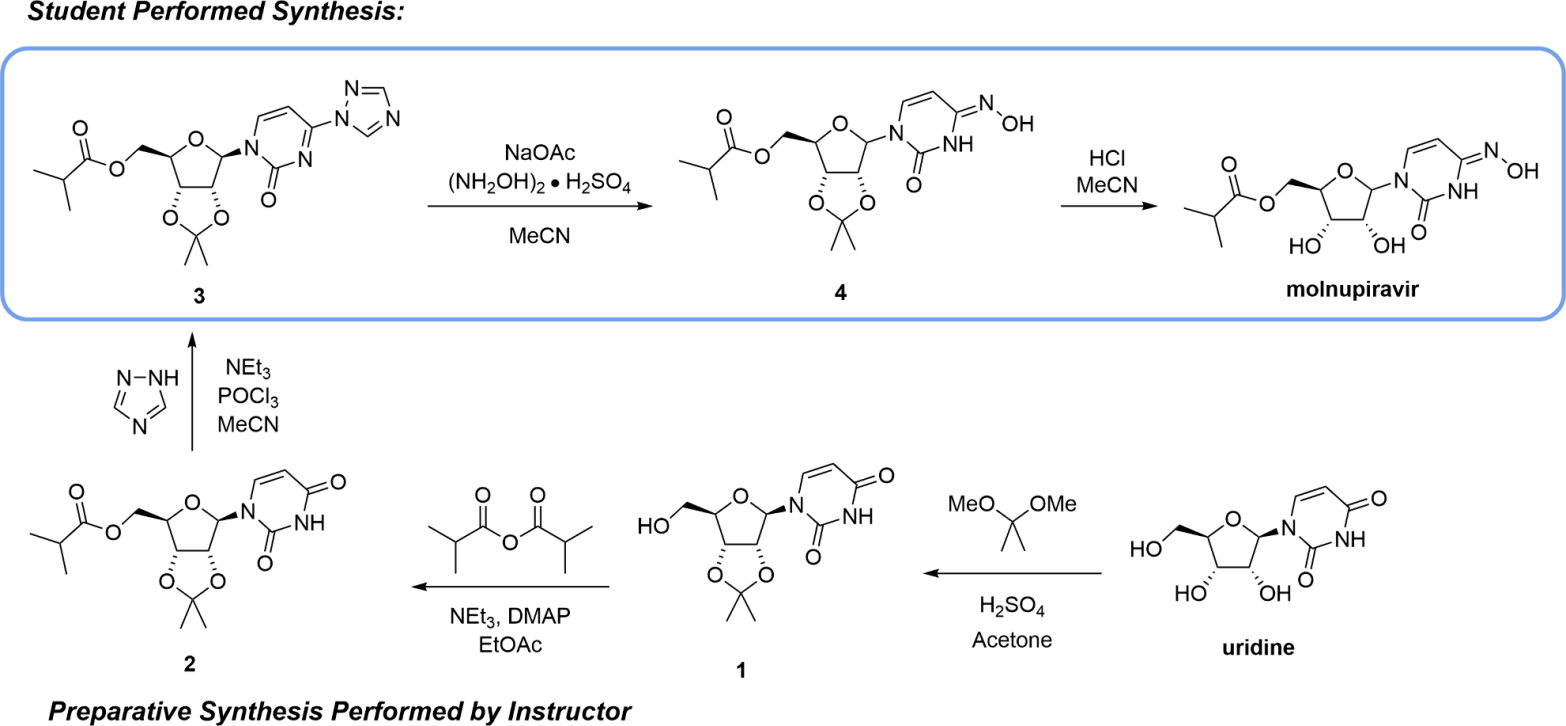
Synthetic Route Used to Prepare Molnupiravir in the Teaching Laboratory, Adapted from Process Literature The students performed the final two steps, highlighted in the blue box. The instructor prepared **3** on a multigram scale prior to student use.

The experimental steps to prepare molnupiravir from uridine were adapted in the first part of the spring 2022 semester by one of the instructors and an undergraduate research student and tested by a second undergraduate research student prior to the class performing the experiment (Scheme 1). Given the time constraints of the semester and the tolerance of ambient atmosphere, the last two steps that convert **3** to molnupiravir were chosen for the students enrolled in the course to perform using material that had been generated by the instructor and the undergraduate research students (see synthetic procedures in the Supporting Information). The instructor and undergraduate research students were able to prepare the needed starting material on a 9.3-g scale working around their respective class schedules over a 3-week period. As each student group in the teaching laboratory used 0.4 g of the starting material, the prepared starting material was enough to supply 23 student groups.

While the students in this laboratory section completed the final two steps in the laboratory, all five steps or a different subsection of the five steps could be performed by undergraduate chemistry students depending on the course context and available facilities and equipment. Steps 1 and 2 to prepare intermediates **1** and **2**, respectively, can easily be performed on a large scale, require relatively inexpensive reagents, and do not require any special handling other than working in a fume hood. Step 3 to prepare intermediate **3** is more technically tricky, requiring anhydrous conditions to produce a high yield. It also requires handling of 1,2,4-triazaole, fine powders of which can be combustible in air and give off noxious fumes. Thus, this step would best be performed by upper-level students in a medicinal chemistry course or a capstone laboratory course with sufficient equipment such as gas manifolds to perform the reaction under inert atmosphere.

In the first week, the students enrolled in the course set up the nucleophilic acyl substitution reaction that converts triazole **3** into oxime **4**. These reaction conditions are modified from the process route to allow the students to handle solid reagents, instead of using concentrated solutions of hydroxylamine.^5^ This reaction mixture was allowed to stir overnight at room temperature; the next morning, the instructor collected the reaction vials and stored them in a −20 °C freezer for the rest of the week until the second laboratory period.

In week 2, the students performed liquid–liquid extraction to isolate their crude product oxime **4** and tested the efficacy of their reactions by thin-layer chromatography (TLC). An aliquot of this product was dissolved in DMSO-*d*_6_ for ^1^H NMR analysis, and another aliquot was dissolved in acetonitrile for high-pressure liquid chromatography (HPLC) analysis; the rest was dried and subjected to acid hydrolysis, which was allowed to stir overnight at room temperature. Similar to the previous week, the instructor came in the next morning and placed all of the reactions in the −20 °C freezer until the next laboratory period.

The temperature at which the hydrolysis of **4** into molnupiravir is run influences the number of hydrolysis byproducts observed. In the first iteration of this experiment module, students allowed their reactions to equilibrate at temperatures between 30 and 70 °C. The highest yield was found for the group whose reaction equilibrated at 40 °C, but students whose reactions equilibrated between 55 and 60 °C also produced molnupiravir as the major product but with byproducts from the hydrolysis of the ester and oxime. Subsequent experimentation by the instructor found that, at room temperature, molnupiravir could be formed as the major product with minimal byproducts, and these conditions were used in the second iteration of the experiment.

In week 3, the students isolated crude molnupiravir via liquid–liquid extraction and analyzed the results by TLC. A portion of each product was dissolved in DMSO-*d*_6_ for ^1^H NMR analysis, and another aliquot was dissolved in acetonitrile for HPLC analysis. Week 4 was devoted to interpretation of the ^1^H NMR and HPLC data, and the postlaboratory assignment was due a week later.

## Hazards

All work should be performed in fume hoods, while wearing safety glasses and nitrile gloves. Organic solvents (acetonitrile, ethyl acetate) are flammable. 1,2,4-Triazole has been shown to be a highly energetic molecule with an exotherm onset at 280 °C, so care should be taken to ensure gentle handling in the conversion of **2** to **3** and **3** to **4**. Molnupiravir is an active pharmaceutical molecule, and its precursors are likely bioactive as well, so these must only be handled while wearing personal protective equipment and in a fume hood. In the event of accidental skin contact with any of the chemicals, the skin should be immediately flushed with water. Hydrochloric acid solutions are corrosive and can cause burns. Accidental contact with clothes may result in the decomposition of the fabric.

## Results and Discussion

**Figure 2:**
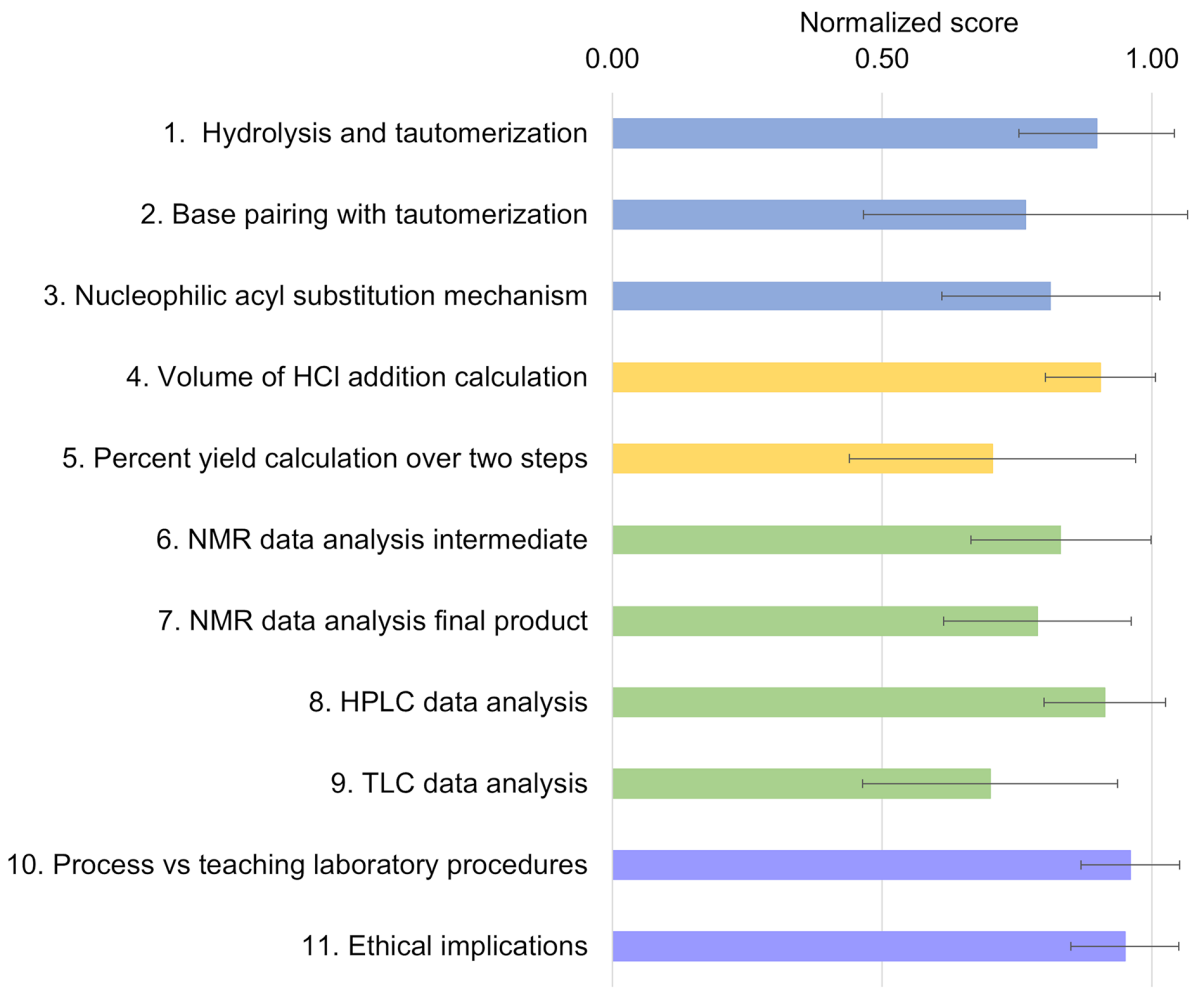
Normalized mean scores and standard deviations (*n* = 24) for postlaboratory assignment questions. The bars are color-coded as a function of question type: application of lecture course material (blue), planning and executing a two-step synthesis (gold), interpretation of experimental data (green), and critical thinking in applying the laboratory content to the broader world (purple).

The laboratory experiment was performed in two consecutive academic years by 30 total students working in 13 groups of 2–3 students each over 4 weeks. Of the 13 groups, all produced oxime **4** in the first step, and 11 groups successfully produced the molnupiravir product as either the largest or second-largest component of their crude product mixture for the second step as determined by HPLC. Students turned in laboratory notebook records for weeks 1–3, but the primary mode of learning assessment was the postlaboratory assignment (see Supporting Information). The questions of the postlaboratory assessment centered on four major themes, performance on which is summarized in Figure 2: (1) application of knowledge from the corequisite organic chemistry lecture course (blue bars); (2) planning and execution of a two-step synthesis (gold bars); (3) interpretation of experimental data (green bars); and (4) critical thinking in the application of science knowledge to the broader world (purple bars).

The laboratory sequence was timed to begin at the midpoint of the semester and run over the weeks that the students were learning carbonyl chemistry: nucleophilic addition, nucleophilic acyl substitution, and carbonyl condensation. Therefore, the postlaboratory assignment asked the students to draw the product of the ester hydrolysis reaction that occurs in vivo and the product of its tautomerization, which most students were able to do with only minor errors, if any errors at all (Figure 2, row 1). Using a figure of RNA base pairing as a starting point, the students used chemical drawing software to show how the two tautomers of the hydrolyzed product could masquerade as uridine and cytidine in base pairing (Figure 2, row 2). The most common error in this question was that some students did not recognize that molnupiravir must tautomerize to bond to adenosine versus guanosine despite being asked to draw this tautomerization in the previous question. The students were also asked to draw the formation of the oxime in step 1 via nucleophilic acyl substitution, which most were able to do with only minor mechanistic errors such as imprecise drawing of curved arrows or mixing up the sequence of resonance and proton transfer (Figure 2, row 3).

For most of the students, this experiment was the first time they had used the product of one reaction as the reactant in a second reaction. In lab during week 2, they were provided with an example calculation of how to scale their second reaction based on the isolated amount of oxime **4**. This activity generated a lot of discussion between students and the instructor, and most students were able to reproduce this calculation on the postlaboratory assessment with either no mistakes or minor mistakes (Figure 2, row 4).

In the course of the laboratory experiment, the students acquired thin-layer chromatography (TLC), HPLC, and ^1^H NMR data after each step. Obtaining authentic characterization data of their own products promotes student accountability and motivates student engagement as compared to when students are simply given example data. These methods were chosen due to the utility of these instruments in assessing compound identity and purity as well as availability of the instrumentation for use by teaching laboratory students. However, for institutions with different available instrumentation, other characterization methods could be used such as mass spectrometry or infrared spectroscopy.

**Figure 3:**
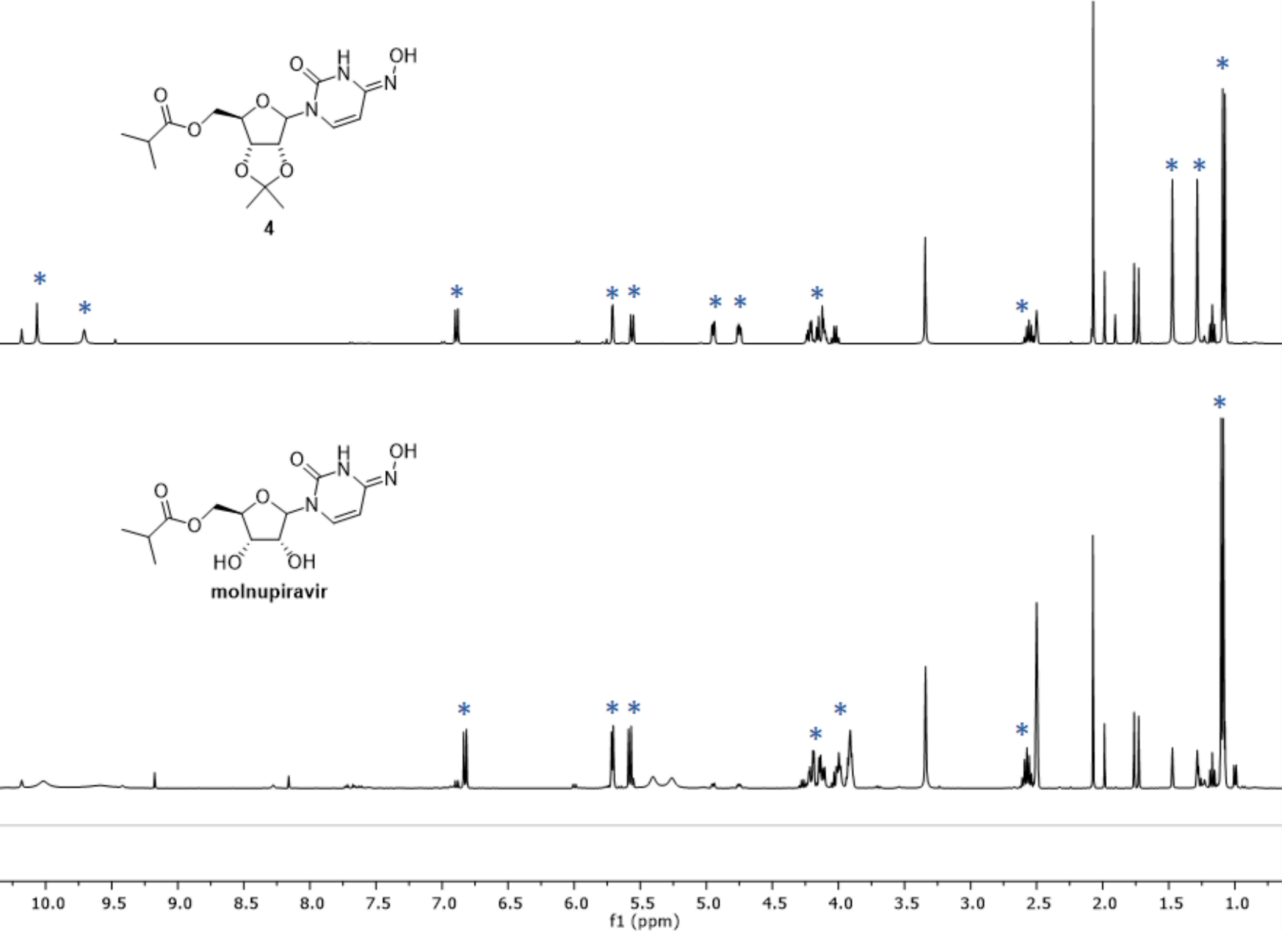
Student ^1^H NMR data of oxime **4** and the molnupiravir final product.

The students already had experience downloading and analyzing ^1^H NMR data on their personal computers from two prior laboratory experiments. They were provided a copy of the supporting information from the referenced paper and asked to use that information to assign the peaks in their ^1^H NMR spectra to confirm whether they had made their desired products in the postlaboratory assignment.^7^Figure 3 shows examples of student obtained ^1^H NMR data for oxime **4** and molnupiravir. For the postlaboratory assignment, the students were asked to present their processed ^1^H NMR spectra and, given the chemical shifts in DMSO-*d*_6_ of the reaction and purification solvents used, to comment on the purity of their products, including whether or not the ^1^H NMR data from the final crude reaction mixture agreed with the HPLC data (Figure 2, rows 6 and 7). Some, but not all, students recognized that the residual solvent that was obvious in their product from step 1 impacted the accuracy of their scale calculation for step 2. They used their determination of purity in their assessment of the accuracy of their percent yield calculation over the two-step reaction sequence (Figure 2, row 5). ^1^H and ^13^C NMR spectra for all synthetic intermediates can be found in the Supporting Information.

**Figure 4:**
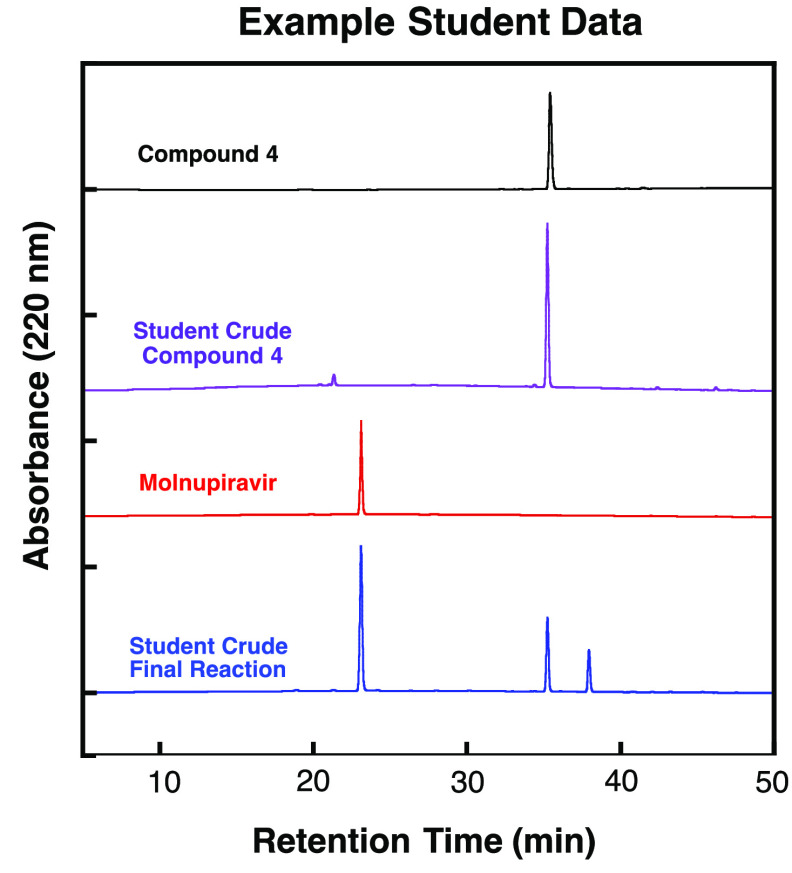
HPLC traces of standards of **4** and molnupiravir as well as example student data of crude reactions during molnupiravir synthesis.

This laboratory experiment was the first introduction of many students to HPLC, so the prelab lecture for the data analysis session in week 4 included an introduction to this technique that drew on their existing knowledge of TLC and column chromatography from the Organic Chemistry I laboratory. They were provided with a table of retention times for the molnupiravir product (Table S1) and synthetic intermediates and shown how to integrate major peaks in their HPLC traces and export their data. Examples of student HPLC data along with those of instructor prepared and purified standards can be found in Figure 4. Despite their relative inexperience with this instrumental technique, most students were able to accurately determine the major product of their reaction mixtures and comment on the relative purity in their postlaboratory assignment (Figure 2, row 8). HPLC chromatograms for all synthetic intermediates and the final molnupiravir product are provided in the Supporting Information (Figure S6). Interestingly, the students struggled more with the postlaboratory assignment question on using their TLC data to discuss how the relative polarity of the molecule changes as it converts from triazole **3** to oxime **4** and finally the liberated diol in the molnupiravir final product (Figure 2, row 9). Some students got mixed up as to how *R*_f_ values relate to molecular polarity, which then complicated their discussions of how the functional group changes impacted the molecular polarity.

Because the reaction setup in the first week was quick, a portion of the remainder of the laboratory period during the first iteration of the experiment was utilized for a video call with a chemist employed in the process chemistry sector of the pharmaceutical industry. The students prepared for this video call by completing a prelaboratory activity in which they read passages from the process chemistry primary literature on the reaction they executed in week 1 and contrasted the scale, techniques, and language to the procedure they employed in the teaching laboratory. The postlaboratory assignment returned to this theme by asking the students to discuss the differences in writing style and jargon between the process chemistry articles on molnupiravir synthesis and the “cookbook” experiments that they typically follow in the teaching laboratory (Figure 2, row 10). In the second year of the course, scheduling constraints did not allow for a similar interaction with a professional process chemist, but the students were still asked to discuss the stylistic differences between the process chemistry literature and the typical teaching laboratory procedure. In both years, students were able to point out multiple differences between the two documents, including the scale, reaction “agitation” versus reaction “stirring”, and changes in heating and cooling times.

Many of the students in this course aspire to enroll in professional health programs, following the receipt of their undergraduate degrees. To tie in the laboratory experiment they had just executed to their career interests, the last question on the postlaboratory assignment in the first iteration of the experiment shared some of the information on the current practices in treating patients with active COVID-19 infections and information from the molnupiravir emergency use authorization document pertaining to the authorized populations for this drug. The students were then asked to reflect on how they would make decisions with regard to treatment options for different populations if they were a physician. By the time of the second iteration of the experiment, there were more studies of the efficacy of molnupiravir and its potential to expedite the development of new variants of the SARS-CoV-2 virus.^18^ Accordingly, in that version of the postlaboratory assignment, students were asked to read the referenced article and to write a paragraph commenting on the relative risks and benefits of prescribing molnupiravir using data from that source. In both years, the instructor was impressed with the level of critical thinking and thoughtfulness displayed by the students as they carefully considered how these important decisions could potentially impact human health on both an individual and societal level (Figure 2, row 11).

## Conclusion

In conclusion, the process chemistry literature on the COVID-19 antiviral drug molnupiravir has been used to design a multistep synthetic chemistry laboratory experiment for second semester organic chemistry students. The laboratory leverages knowledge of carbonyl chemistry learned concurrently in the lecture section and continues to develop students’ analytical skills in characterization of their products via ^1^H NMR and HPLC. The laboratory is also used as an opportunity to introduce students to chemistry in industry, with a focus on what process chemistry is and how it differs from the typical laboratory experiments that students have encountered in the teaching laboratory to date.

## Supporting Information Available

Student handout and postlaboratory assignment (PDF; DOCX)
Additional synthetic information; tables and figures related to HPLC, ^1^H NMR, and ^13^C NMR spectra of intermediates **1**–**4** and molnupiravir (PDF)
