# Supplementary material for: Scaling the Process Chemistry of a COVID-19 Antiviral Pharmaceutical Down for a Multistep Synthesis Experiment in the Undergraduate Teaching Laboratory
Source: J Chem Educ. 2024 Feb 7;101(3):1211–7. doi: 10.1021/acs.jchemed.3c00999 (PMC10938635; doi:10.1021/acs.jchemed.3c00999)
Supplement: Supplementary file 2 — ed3c00999_si_002.docx [file ed3c00999_si_002.docx]

Supporting Information: Laboratory experiment handout and post-laboratory assignment

for

**Scaling the Process Chemistry of a COVID-19 Antiviral Pharmaceutical Down for a Multi-Step Synthesis Experiment in the Undergraduate Teaching Laboratory**

Andrew J. Wommack, ^‡ †^ Aaliyah B. Holloway, ^†^ Kaitlyn A. Stallings, ^†^ and Pamela M. Lundin*^†^

^†^ Department of Chemistry, High Point University, High Point, North Carolina 27268, United States

^‡^  Cambrex, High Point, North Carolina 27265, United States

*Email: [plundin@highpoint.edu](mailto:plundin@highpoint.edu)

# The Synthesis of Molnupiravir Purpose

1. To perform multi-step synthesis of an organic molecular target.
2. To practice determining reaction conversion and success using NMR spectral and HPLC data.
3. To compare and contrast the language used in process chemistry protocols with that used in the teaching laboratory.

# Background

Molnupiravir is an anti-viral small molecule pharmaceutical drug that was authorized for emergency used by the FDA as a treatment for COVID-19 in December 2021 (Figure 1).1 Originally developed by Ridgeback Biotherapeutics and licensed for scale-up and production by Merck, molnupiravir is structurally very similar to the nucleosides cytidine and uridine (Figure 1). In the body, the ester is hydrolyzed to the primary alcohol and reacts to form a triphosphate, similar to the nucleic acids that are incorporated into RNA. The hydroxyamine group tautomerizes to mimic its nucleoside analogs and is incorporated into viral genome during RNA replication, but because it is not actually cytidine or uridine, it halts replication, thus preventing the virus from proliferating in its host.2


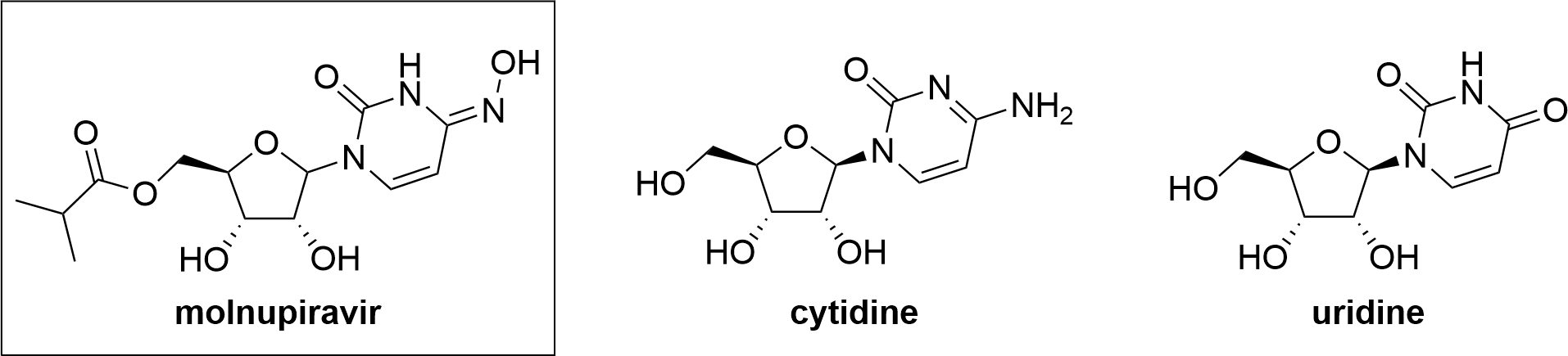


**Figure S1:** Comparison of structures of molnupiravir, cytidine, and uridine

Molnupiravir is not accessible by isolation from natural sources; it must be synthesized via multi- step synthesis. Producing a drug molecule on a scale of thousands of kilograms for distribution brings specific challenges that must be considered. How can you design a synthesis that uses very cheap precursors that are readily available and can lead to your desired target as fast as possible, as cheap as possible, and with the highest yield possible? What waste is generated, what is its environmental threat and disposal cost? If the reaction occurs faster in one area of reactor for a multi-kilogram reaction, a hot spot could develop leading to runaway reactions, so how is the temperature controlled and how is appropriate mixing ensured?

In this multi-week laboratory experiment, we are going to follow a process synthesis procedures of molnupiravir that has been adapted for the teaching laboratory scale (Scheme 1).3,4 This is a five- step process that would take you the entire semester to perform if you were to start at the beginning. We have already performed reactions and purification for intermediates **1**-**3**; you will spend the next several weeks performing the last two steps to make **4** and molnupiravir.

We will lead you through the synthetic procedures as we normally do in the teaching laboratory, but we will also look at the text of the paper and think about how the language used in the teaching laboratory differs from that used for the process scale. Additionally, we will consider other published process-scale procedures for molnupiravir synthesis and compare and contrast the relative merits and trade-offs of those compared to the one that we are following.


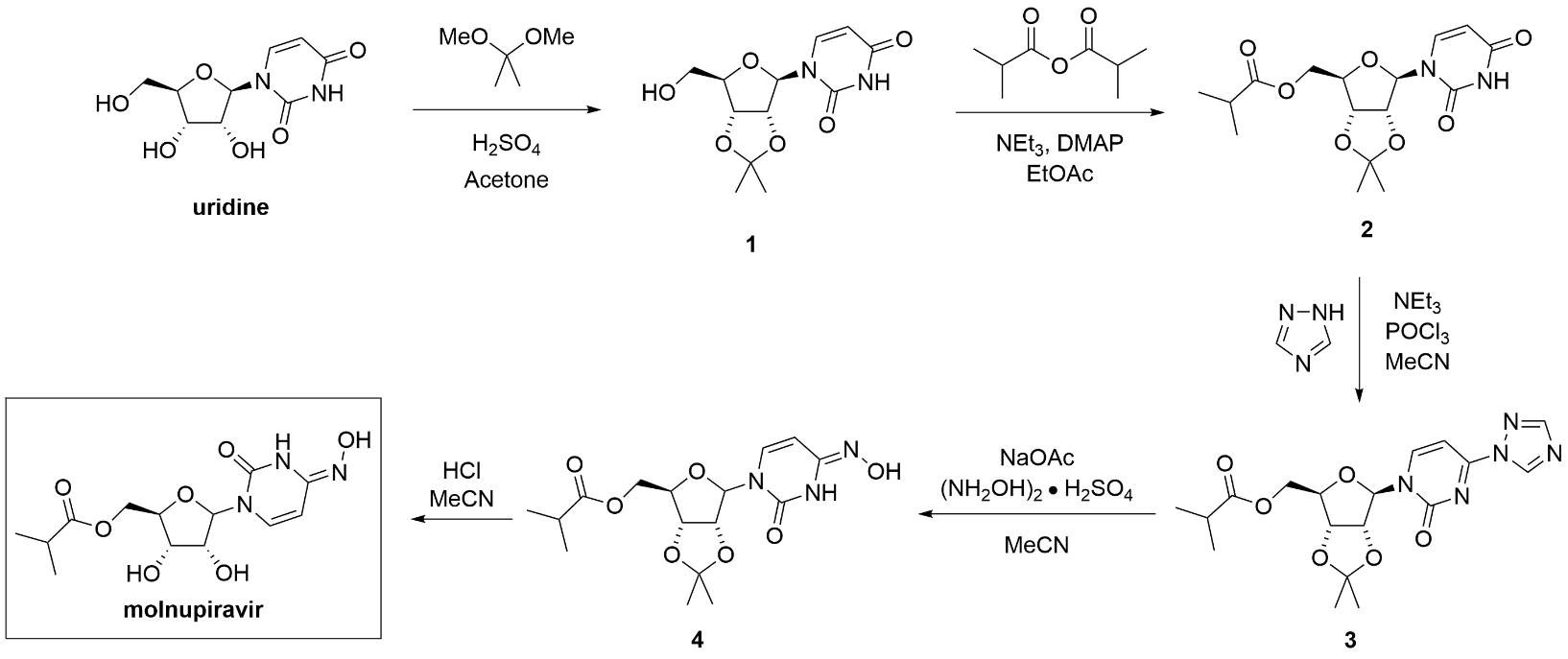


**Scheme S1:** Process synthesis of molnupiravir

Week 1: Pre-Lab Assignment

On Blackboard, there is a document titled “Fier et al. Org. Proc. Res. Dev. 2021”. Read the highlighted text for step 4 on page 12. Then, prepare your notebook for the procedure we will do in lab today using the procedure in this handout. As you do so, compare and contrast the. two procedures for the same reaction. A question to this effect will be on your pre-lab quiz.

# Safety Issues

- 1,2,4-triazole has shown to be a highly energetic molecule with an exotherm onset at 280 °C. Although the risk is low, treat the triazole-containing molecule with care.
- *You are synthesizing an active pharmaceutical molecule.* Handle the organic and inorganic materials only while wearing gloves and goggles, and use them in the hood. In the event that you have accidental skin contact with any of the chemicals, immediately flush the area with water.
- Hydrochloric acid solutions are corrosive and can cause burns. Accidental contact with clothes may result in decomposition of the fabric. Report any contact with skin or eyes to your lab instructor *immediately*.
- All reactions should be done in the hood using proper protective equipment.


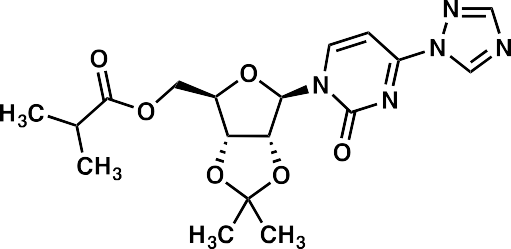

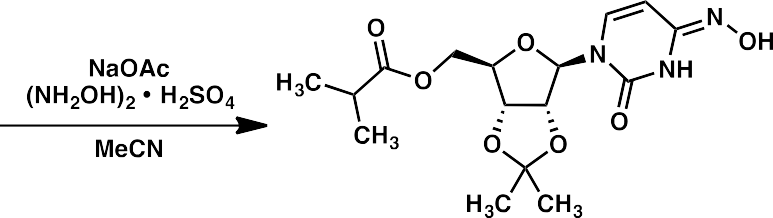


**Figure S2**. Hydoxylamine substitution of triazole following sodium acetate interaction with hydroxylamine sulfate

Reaction Assembly for Oxime Installation

1. Add 0.40 g (±10% but record your mass!) of the starting triazole-containing molecule to a provided glass vial with a Teflon- coated stir bar.
2. Next, measure 0.40 g of the hydroxylamine sulfate salt on weigh paper and add it to the reaction mixture.
3. Measure 0.20 g of sodium acetate on weigh paper and add it to the reaction mixture.
4. To this solid mixture, add 3.0 mL of acetonitrile (MeCN) to the vial and use the magnetic stir plate to stir the slurry.
5. Securely affix the vial cap and continue stirring at ambient temperature. Your instructor will place your reaction in the freezer tomorrow, and it will stay there until next week.

# Cleaning Up

Make sure that all surfaces are wiped down with paper towels. The salts used in this lab are hygroscopic and are corrosive. *Make sure the balance area is clean!!!*

# Procedure: Week 2

Reaction Work-Up

1. After the >16-hr reaction period, dilute the reaction with deionized water (ca. 4 mL) and add ethyl acetate (ca. 4 mL).
2. Stir the biphasic mixture within the reaction vial using the stir bar and stir plate. Then, let the contents separate.
3. Prepare a TLC plate with 1) a spot of starting triazole, 2) your crude reaction mixture (ethyl acetate layer from previous step, and 3) a co-spot of each. **Take care to not cross contaminate by washing your TLC spotter. ** For the TLC mobile phase, use 3:1 Ethyl Acetate/Hexanes. Record your data in some form.
4. Transfer the biphasic reaction vial contents to a separatory funnel with ethyl acetate rinses of the reaction vial.
5. Add ca. 40 mL of water and ca. 40 mL of ethyl acetate to the separatory funnel and perform a standard extraction.
6. Drain the aqueous layer in a beaker and then collect the organic layer in a clean Erlenmeyer flask.
7. Add sodium sulfate to dry the collected organic solution and filter the solution to collect the organic filtrate into a clean and pre-weighed 100-mL round bottom flask.
8. Use the rotovap to remove the volatile organics and solvent to deliver the reaction product. Measure the crude isolated product amount by mass difference and determine your reaction yield.
9. Add 12 mL of MeCN to the crude product to re-dissolve. Take an aliquot of this solution (ca. 2-3 mL) and place it in a clean scintillation vial.
10. Using a pipettor, take a 100-μL aliquot from this MeCN solution and place it in an HPLC vial. Dilute this aliquot with 900 μL of 1:1 MeCN/water and cap the HPLC vial. Use a sharpie to label your vial and submit the sample for data acquisition on analytical HPLC.
11. Use the Rotovap to remove the remainder of MeCN from the scintillation vial. Use DMSO-d6 to prepare a NMR sample of your reaction product aliquot.


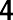


**Figure S3**. Acidic dimethyl acetal hydrolysis in acetonitrile

Reaction Assembly for Dimethylacetal Hydrolysis


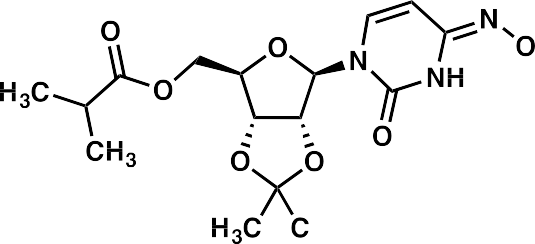

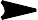

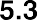

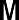

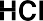

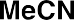

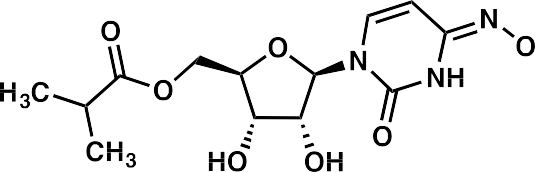


1. Use the Rotovap to remove MeCN from the 100-mL round-bottom flask and determine the amount of oxime-functionalized product.
2. Use the reaction conditions given below to calculate the reactant and solvent amounts for your reaction scale (based on your amount of oxime-containing starting material from the previous step).

**Table S1**: Example reactions conditions for scaling reaction

| Chemical Name | MW | Density | Amount | mmol | equiv | source |
| --- | --- | --- | --- | --- | --- | --- |
| Intermediate 4 | 369.1536 | n/a | 0.1398 g | 0.3787 | 1.00 | Notebook 17, page  16 |
| HCl (5.3 M in water) | n/a | 1.065 g/mL | 0.0605 mL | 0.321 | 0.85 | Using deionized  water |
| Acetonitrile | 41.05 | 0.786 | 1.11 mL | 0.34 M | | Solvent  bottle |

1. Add a stir bar to the 100-mL round-bottom flask and then add acetonitrile.
2. Using a pipettor, add the HCl solution to the stirring acetonitrile reaction and fit a ground-glass stopper in the neck of the flask. Let the reaction stir at room temperature overnight. Your instructor will place your reaction in the freezer tomorrow, and it will stay there until next week.

# Cleaning Up

Make sure your work areas and all glassware have been cleaned.

# Week 3: Pre-Lab Assignment

Read the sections on flash chromatography and high performance liquid chromatography in *Making the Connections^3^: A How-To Guide for Organic Chemistry Lab Techniques* by Anne. B. Padias. In edition 3, these sections can be found on pages 180-181. This content will be included in the pre-lab quiz.

# Procedure: Week 3

1. Dilute the reaction with deionized water (ca. 4 mL) and add ethyl acetate (ca. 4 mL).
2. Stir the biphasic mixture within the reaction flask using the stir bar and stir plate. Then, let the contents separate.
3. Prepare a TLC plate with 1) a spot of starting triazole, 2) your crude reaction mixture (ethyl acetate layer from previous step, and 3) a co-spot of each. **Take care to not cross contaminate by washing your TLC spotter. ** For the TLC mobile phase, use 3:1 Ethyl Acetate/Hexanes.
4. Transfer the biphasic reaction contents to a separatory funnel with ethyl acetate rinses of the reaction vial.
5. Add ca. 20 mL of water, ca. 20 mL of sodium bicarbonate solution, and ca. 40 mL of ethyl acetate to the separatory funnel and perform a standard extraction.
6. Drain the aqueous layer in a beaker and then collect the organic layer in a clean Erlenmeyer flask.
7. Add sodium sulfate to dry the collected organic solution and filter the solution to collect the organic filtrate into a clean and pre-weighed 100-mL round bottom flask.
8. Use the rotovap to remove the volatile organics and solvent to deliver the crude reaction product. Measure this isolated product amount by mass difference and determine your reaction yield.
9. Add 12 mL of MeCN to the crude product to re-dissolve. Take an aliquot of this solution (ca. 2-3 mL) and place it in a clean scintillation vial.
10. Using a pipettor, take a 100-μL aliquot from the MeCN solution and place it in an HPLC vial. Dilute this aliquot with 900 μL of 1:1 MeCN/water and cap the HPLC vial. Use a sharpie to label your vial and submit the sample for data acquisition on analytical HPLC.
11. Use the Rotovap to remove the MeCN from the scintillation vial. Use DMSO-d6 to prepare a NMR sample of your reaction product aliquot.
12. Transfer the remaining product solution from the round bottom flask to a second clean, tared scintillation vial. Write your name and your partner’s name on the vial along with “Molnupiravir product week 3”. Store the vial with the class samples.

# Cleaning Up

Make sure your work areas and all glassware have been cleaned.

# Week 4: Pre-Lab Assignment

Draw the structures of starting material **3**, product **4** and **molnupiravir**. Make notes about what ^1^H NMR signals will be indicative of the structural changes between these three molecules. Also note which structural changes will results in increases in polarity and which will result in decreases in polarity.

# Procedure: Week 4

1. Download your ^1^H NMR spectra from weeks 2 and 3. Compare your ^1^H NMR data to that reported in the *Org. Proc. Res. Dev.* paper by Fier et al.
2. With your group, load your HPLC data and analyze it by comparing it to your standards. Take a screenshot of each of your spectra and save these to a flash drive for use in your post-lab.

**References**

1. Commissioner, O. of the. Coronavirus (COVID-19) Update: FDA Authorizes Additional Oral Antiviral for Treatment of COVID-19 in Certain Adults https://[www.fda.gov/news-](http://www.fda.gov/news-) events/press-announcements/coronavirus-covid-19-update-fda-authorizes-additional-oral- antiviral-treatment-covid-19-certain (accessed 2022 -02 -17).
2. Kabinger, F.; Stiller, C.; Schmitzová, J.; Dienemann, C.; Kokic, G.; Hillen, H. S.; Höbartner, C.; Cramer, P. Mechanism of Molnupiravir-Induced SARS-CoV-2 Mutagenesis. *Nat. Struct. Mol. Biol.* **2021**, *28* (9), 740–746. https://doi.org/10.1038/s41594-021-00651-0.
3. McIntosh, J. A.; Benkovics, T.; Silverman, S. M.; Huffman, M. A.; Kong, J.; Maligres, P. E.; Itoh, T.; Yang, H.; Verma, D.; Pan, W.; Ho, H.-I.; Vroom, J.; Knight, A. M.; Hurtak, J. A.; Klapars, A.; Fryszkowska, A.; Morris, W. J.; Strotman, N. A.; Murphy, G. S.; Maloney, K. M.; Fier, P. S. Engineered Ribosyl-1-Kinase Enables Concise Synthesis of Molnupiravir, an Antiviral for COVID-19. *ACS Cent. Sci.* **2021**, *7* (12), 1980–1985. https://doi.org/10.1021/acscentsci.1c00608.
4. Fier, P. S.; Xu, Y.; Poirier, M.; Brito, G.; Zheng, M.; Bade, R.; Sirota, E.; Stone, K.; Tan, L.; Humphrey, G. R.; Chang, D.; Bothe, J.; Zhang, Y.; Bernardoni, F.; Castro, S.; Zompa, M. A.; Taylor, J.; Sirk, K. M.; Diaz-Santana, A.; Diribe, I.; Emerson, K. M.; Krishnamurthi, B.; Zhao, R.; Ward, M.; Xiao, C.; Ouyand, H.; Zhan, J.; Morris, W. J. Development of a Robust Manufacturing Route for Molnupiravir, an Antiviral for the Treatment of COVID-19. *Org. Process Res. Dev.* **2021**, *25* (12), 2806–2815. https://doi.org/10.1021/acs.oprd.1c00400.

# Post-Lab Assignment: The Synthesis of Molnupiravir

1. In the body, the ester in molnupiravir is hydrolyzed, and then the resulting alcohol is phosphorylated with a triphosphate group to form the active pharmaceutical structure. Draw a reaction scheme showing molnupiravir hydrolysis of the ester, followed by tautomerization of the oxime.
2. The following figure shows how uridine base pairs with adenosine and cytidine base pairs with guanidine. The tautomerization of the hydrolyzed molnupiravir allows its positioning of lone pairs and hydrogens to mimic the hydrogen bonding abilities of both uridine and cytidine. Thus it is incorporated into the viral genome, but then begins scrambling the sequence (i.e. a mutation), leading to halting of replication after 4 or 5 induced mutations.

Show how this incorporation is possible by editing the ChemDraw figure below to replace uridine and cytidine with the appropriate tautomer of molnupiravir in the base pairing scheme.

**Figure S4:** editable ChemDraw figure for showing molnupriavir tautomers in base pairing.

1. In week 1, you set up a substitution reaction wherein the hydroxyamine performs a nucleophilic acyl substitution-type mechanism to expel the triazole leaving group. Using ChemDraw, draw the mechanism of this reaction.
2. You collected TLC data to monitor your reaction progress in weeks 2 and 3. Insert a ChemDraw figure of triazole starting material **3**, synthetic intermediate **4**, and final molnupiravir product, annotated to show areas of polarity in each molecule. Then present the Rf data that you collected (the form could be a table, pictures, or cartoons) and discuss the relative polarity of the triazole starting material **3**, synthetic intermediate **4**, and final molnupiravir product.
3. Insert a figure of your HPLC traces from weeks 2 and 3 and annotate these figure to show the **4** and the molnupiravir desired product and identify the byproducts. Based on this data, how pure is your product? Considering that this HPLC is a reverse phase HPLC, meaning more polar molecules elute first, does this data correlate to your TLC data?
4. Insert your ^1^H NMR spectrum of synthetic intermediate **4** along with an annotated structure of **4**. Discuss how well your ^1^H NMR data match the literature data reported in the *Org. Proc. Res. Dev.* paper and the relative purity that you observe of this product.

*Note: reference the DMSO-d_6_ to 2.50 ppm. In this solvent, the CH_3_ from acetonitrile shows up as a singlet at 2.07 ppm, and the peaks from ethyl acetate are at 1.99 ppm (s, 3H), 4.06 ppm (q, 2 H), and 1.17 ppm (t, 3H).*

1. Show your calculations that you performed in week 2 based on the table to determine the appropriate amount of HCl to add. Comment on what you did to scale the solvent of acetonitrile used, and why the precision of this calculation is less important than that of the HCl addition.
2. Insert your ^1^H NMR spectrum of the molnupiravir final product along with an annotated structure of molnupiravir. Discuss how well your ^1^H NMR data matches the literature data reported in the *Org. Process Res. Dev.*  paper. Comment on the relative purity indicated by your 1H NMR data and how that compares to the purity determined by HPLC.

*Note: reference the DMSO-d_6_ to 2.50 ppm. In this solvent, the CH_3_ from acetonitrile shows up as a singlet at 2.07 ppm, and the peaks from ethyl acetate are at 1.99 ppm (s, 3H), 4.06 ppm (q, 2 H), and 1.17 ppm (t, 3H).*

1. Show your calculation of your percent yield over the two steps of this reaction. Based on your HPLC data and 1H NMR data, how accurate is this percent yield value? Explain.
2. We have discussed how in addition to reaction efficacy, reagent cost and waste generation are also important considerations in the teaching lab and when scaling up a synthetic procedure for commercial production. In a short paragraph (~3-4 sentences) comment on how the procedures we utilized in this laboratory compare to the procedures reported in the *Org. Process Res. Dev.* paper. For your convenience, a pdf with the relevant passages highlighted has been posted on Blackboard.
3. Molnupiravir has recently been in the news, as some speculate that the drug can actually result in new COVID-19 viral variants, especially in immunocompromised individuals whose bodies have trouble clearing the virus. Read the article (doi [10.1126/science.adg9677](http://www.doi.org/10.1126/science.adg9677)) and write a 3-5 sentence paragraph weighing the relative risks and benefits in prescribing molnupiravir to different populations of patients. In your paragraph, be sure to utilize information and data from the article.
