# Supplementary material for: Scaling the Process Chemistry of a COVID-19 Antiviral Pharmaceutical Down for a Multistep Synthesis Experiment in the Undergraduate Teaching Laboratory
Source: J Chem Educ. 2024 Feb 7;101(3):1211–7. doi: 10.1021/acs.jchemed.3c00999 (PMC10938635; doi:10.1021/acs.jchemed.3c00999)
Supplement: Supplementary file 3 — ed3c00999_si_003.pdf [file ed3c00999_si_003.pdf]

Supporting Information for:

Scaling the Process Chemistry of a COVID-19 Antiviral Pharmaceutical Down for a Multi-Step Synthesis Experiment in the Undergraduate Teaching Laboratory

Andrew J. Wommack,[‡ †] *Aaliyah B. Holloway,*[†] *Kaitlyn A. Stallings,*[†] *and Pamela M. Lundin**[†]

[†] Department of Chemistry, High Point University, High Point, North Carolina 27268, United States

[‡] Cambrex, High Point, North Carolina 27265, United States

*Email: plundin@highpoint.edu

Table of Contents

1. Materials and Equipment S-1
2. Procedures and Methods S-2
3. Analytical HPLC S-7
4. ^{1}H and ^{13}C NMR Data S-8

1. Materials and Equipment

The following suppliers were used for key reagents: uridine (TCI), anhydrous acetone (Acros), 2,2-dimethoxypropane (Alfa Aesar), triethylamine (Sigma-Aldrich), 4-dimethylaminopyridine (Aldrich), isobutyric anhydride (TCI), 1,2,4-triazole (Beantown Chemical), phosphorus oxychloride (Beantown Chemical), hydroxylamine sulfate (TCI), sodium acetate (Alfa Aesar).

NMR spectra were recorded using a JEOL ECZ-400S NMR spectrometer. HPLC was acquired on a Shimadzu Nexera LC-40 with a photodiode array detector. Flash chromatography was performed on a Biotage Seleckt flash purification system.

2. Procedures and Methods

All synthetic procedures were adapted from reactions previously described in the process scale literature.[1]

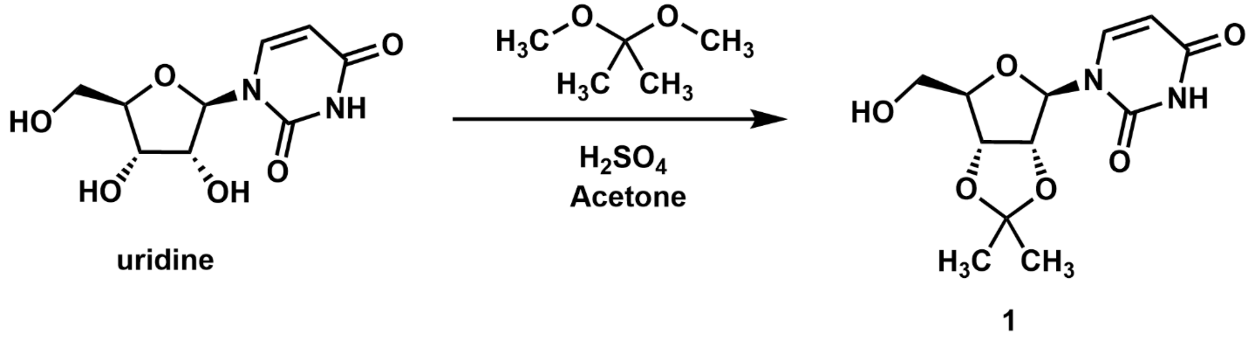

Figure S1: Synthesis of acetal **1** from uridine.

To a flame-dried 100-mL two-neck round bottom flask with a Teflon-coated stir bar under N_2 atmosphere was added uridine (4.00 g, 16.38 mmol, 1.00 equiv). Anhydrous acetone (16.0 mL) was added via gas tight syringe followed by 2,2-dimethoxypropane (4.10 mL, 33.38 mmol, 2.00 equiv). The reaction mixture was not homogenous at this stage. Sulfuric acid (32.0 mg, 0.164 mmol, 0.10 equiv) was added to the reaction using a glass pipette by opening one septum while an active N_2 inlet was connected to the other ground-glass joint. The interior of the glass pipette was washed with anhydrous acetone (4.0 mL) and the rubber septum re-affixed. There was immediate yellow coloration noticed upon addition of the sulfuric acid, which was followed by complete dissolution of the reaction mixture. The clear solution was then allowed to stir gently at ambient temperature (ca. 22 °C) overnight. Following the 16-hr reaction period, the product had precipitated out of the solution. The inert atmosphere was removed, the large precipitate solids were broken into small particles, heptanes (8.0 mL) was added to ensure adequate stirring, and the flask was cooled to 4 °C in an ice bath. At the lower temperature, triethylamine (0.30 mL) was added to neutralize the sulfuric acid. The solid was collected by filtration with heptane washes to deliver product. After vacuum drying Compound **1** was isolated as an ivory powder in 97.1% yield (4.52 g, 15.91 mmol) that matched ^{1}H and ^{13}C NMR from the literature.[1]

^{1}H NMR (400 MHz, DMSO-*d6*) δ ppm 1.29 (s, 3 H), 1.49 (s, 3 H), 3.57 (m, 2 H), 4.07 (q, *J*=4.27 Hz, 1 H), 4.74 (dd, *J*=6.27, 3.51 Hz, 1 H), 4.90 (dd, *J*=6.27, 2.51 Hz, 1 H), 5.08 (t, *J*=5.40 Hz, 1 H), 5.64 (dd, *J*=8.03, 1.25 Hz, 1 H), 5.83 (d, *J*=2.76 Hz, 1 H), 7.79 (d, *J*=8.03 Hz, 1 H), 11.38 (br s, 1 H). ^{13}C NMR (100 MHz, DMSO-*d6*) δ ppm 25.19, 27.05, 61.26, 80.47, 83.67, 86.52, 91.11, 101.73, 112.96, 141.96, 150.34, 163.21.

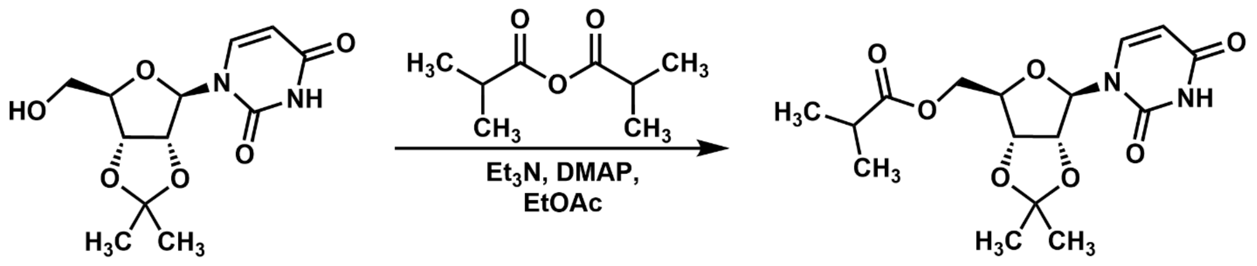

1 **2**

Figure S2: Synthesis of ester **2** from acetal **1**.

To a flame-dried 250-mL single-neck round bottom flask with a Teflon-coated stir bar under N_2 atmosphere was added Compound **1** (9.26 g, 32.59 mmol, 1.00 equiv) as a solid, followed by addition of ethyl acetate (98.0 mL). Next, the reaction flask was set in a 4 °C ice bath before triethylamine (6.80 mL, 48.79 mmol, 1.50 equiv) was added down the sidewalls of the cooled flask, followed by addition of 4-dimethylaminopyridine (79.4 mg, 0.650 mmol, 0.020 equiv). Isobutyric anhydride (5.95 mL, 35.90 mmol, 1.10 equiv) was added dropwise to the center of the rapidly stirring heterogeneous solution. Following the completion of this slow addition, the ice bath was removed for slow warming to ambient temperature (ca. 22 °C) for a 2-hour stirring period. The reaction was observed to be homogeneous after ca. 10 min at the elevated temperature. After the 2-hour reaction time concluded, water (100 mL) was added, the reaction was further diluted with ethyl acetate (50 mL), and the reaction contents were transferred to a separation funnel for washing of the organic layer. The ethyl acetate layer was washed with NH_4Cl saturated aqueous solution (2x 100 mL), once again with water (100 mL), and once with brine (100 mL). The organic layer was collected and dried with Na_2SO_4, filtered and volatiles were removed under reduced pressure to leave Compound **2** as white solid in 98.2% yield (11.33 g, 32.01 mmol) that matched ^{1}H and ^{13}C NMR from the literature.[1]

^{1}H NMR (400 MHz, DMSO-*d6*) δ ppm 1.04 (d, *J*=6.78 Hz, 6 H), 1.25 (s, 3 H), 1.45 (s, 3 H), 4.17 (m, 3 H), 4.75 (dd, *J*=6.27, 3.26 Hz, 1 H), 5.01 (dd, *J*=6.53, 1.76 Hz, 1 H), 5.60 (d, *J*=8.03 Hz, 1 H), 5.75 (d, *J*=1.76 Hz, 1 H), 7.66 (d, *J*=8.03 Hz, 1 H), 11.41 (s, 1 H).
^{13}C NMR (100 MHz, DMSO-*d6*) δ ppm 18.77, 25.17, 26.98, 33.10, 63.86, 80.78, 83.73, 84.32, 92.66, 101.76, 113.29, 142.97, 150.31, 163.32, 175.94.

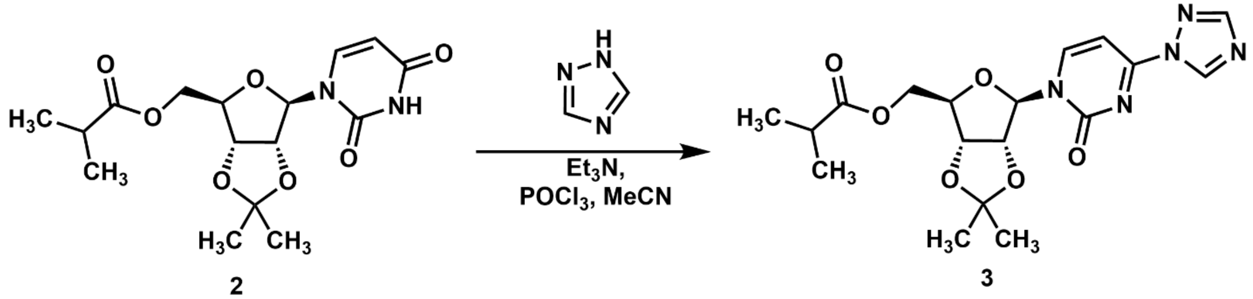

Figure S3: Synthesis of triazole **3** from ester **2**.

To a flame-dried 250-mL single-neck round bottom flask with a Teflon-coated stir bar under N_2 atmosphere was added 1,2,4-triazole (9.80 g, 141.9 mmol, 5.00 equiv) as a solid followed by addition of MeCN (60 mL) with gentle stirring. Triethylamine (36.40 mL, 261.1 mmol, 7.00 equiv) was added and then the reaction was cooled to 4 °C with an ice bath. Phosphorus oxychloride (3.65 mL, 38.93 mmol, 1.30 equiv) was added dropwise and thick slurry formed over 10 min. Next, **2** (9.80 g, 27.67 mmol, 1.00 equiv) was added as a solution in MeCN (40 mL) via cannula addition with N_2 push down the sidewalls of the cooled reaction flask. This was followed by a rinse of the Compound **2** flask with MeCN (30 mL) to ensure complete cannula transfer. The reaction was then set to gentle heating at 45 °C for 18 hr. Upon completion of the reaction period and cooling to ambient temperature, the Et_3N • HCl salts were removed by filtration, and the waste filter cake was washed with ethyl acetate. The volatile organics were removed under reduced pressure to afford the crude product solid that was further purified by silica column chromatography (Using ethyl acetate/heptanes (3:1, *v/v*), Compound **2** has $R_f = 0.45$ and Compound **3** has $R_f = 0.30$) to afford the desired Compound **3** as an ivory solid in 84.2% yield (9.44 g, 23.30 mmol) that matched ^{1}H and ^{13}C NMR from the literature.[1]

^{1}H NMR (400 MHz, DMSO-*d*$_6$) δ 0.94 (dd, *J* = 8.3, 7.0 Hz, 7H), 1.24 (s, 3H), 1.44 (s, 3H), 4.21 (qd, *J* = 12.0, 4.9 Hz, 2H), 4.40 (dd, *J* = 6.2, 2.9 Hz, 1H), 4.76 (dd, *J* = 6.2, 3.2 Hz, 1H), 5.00 (dd, *J* = 6.2, 1.5 Hz, 1H), 5.82 (s, 2H), 6.92 (d, *J* = 7.2 Hz, 1H), 8.35 (s, 1H), 8.41 (d, *J* = 7.4 Hz, 1H), 9.40 (s, 1H). ^{13}C NMR (100 MHz, DMSO-*d*6) δ ppm 18.55, 18.74, 25.09, 26.91, 33.09, 64.18, 81.02, 84.73, 85.70, 93.89, 95.75, 112.83, 143.86, 150.17, 153.62, 154.29, 159.15, 175.79

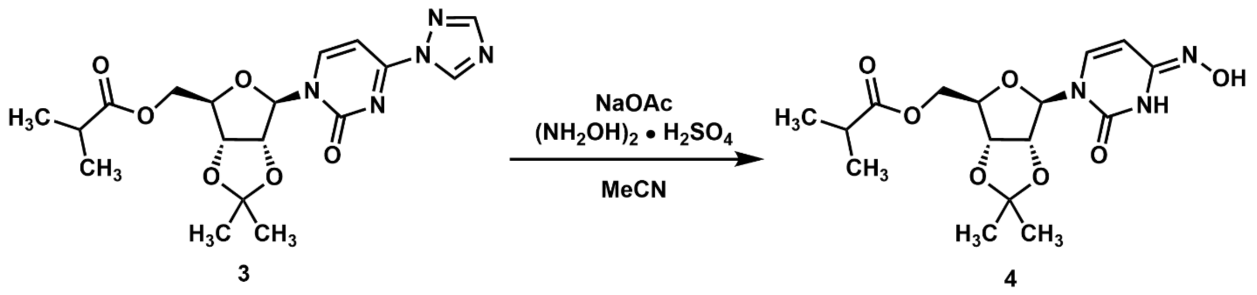

Figure S4: Synthesis of oxime **4** from triazole **3**.

To a 25-mL round-bottom flask with Teflon-coated stirring bar under ambient atmosphere, was added Compound **3** (0.400 g, 0.987 mmol, 1.00 equiv) and acetonitrile (3 mL) was added. After complete dissolution with gentle stirring, hydroxylamine sulfate (0.400 g, 2.437 mmol, 2.47 equiv) and sodium acetate (0.200 g, 2.437 mmol, 2.47 equiv) were added and the suspension was stirred for 18 h at ambient temperature (ca. 22 °C). After the reaction period concluded, the reaction contents were transferred to a separation funnel with ethyl acetate rinses for aqueous washing of the organic layer. The ethyl acetate layer (ca. 40 mL) was washed with water (2x 40 mL) and once with brine (40 mL). The organic layer was shown to contain a single major product using silica TLC. (Using ethyl acetate/heptanes (3:1, *v/v*), Compound **4** has R_f = 0.45 and Compound **3** has R_f = 0.30.) The organic layer was collected and dried with Na_2SO_4, filtered, and volatiles were removed under reduced pressure to leave Compound **4** as white solid in 90.6% yield (0.330 g, 0.894 mmol) that matched ^{1}H and ^{13}C NMR from the literature.[1]

^{1}H NMR (400 MHz, DMSO-*d6*) δ ppm 1.04 (d, *J*=7.03 Hz, 6 H), 1.24 (s, 3 H), 1.43 (s, 3 H), 4.11 (m, 3 H), 4.71 (dd, *J*=6.27, 4.02 Hz, 1 H), 4.91 (dd, *J*=6.53, 2.26 Hz, 1 H), 5.52 (d, *J*=8.03 Hz, 1 H), 5.67 (d, *J*=2.26 Hz, 1 H), 6.85 (d, *J*=8.28 Hz, 1 H), 9.68 (s, 1 H), 10.04 (s, 1 H). ^{13}C NMR (100 MHz, DMSO-*d6*) δ ppm 18.82, 25.22, 27.05, 33.11 63.91, 80.62, 83.18, 91.31, 98.73, 113.44, 132.04, 143.33, 149.15, 176.00.

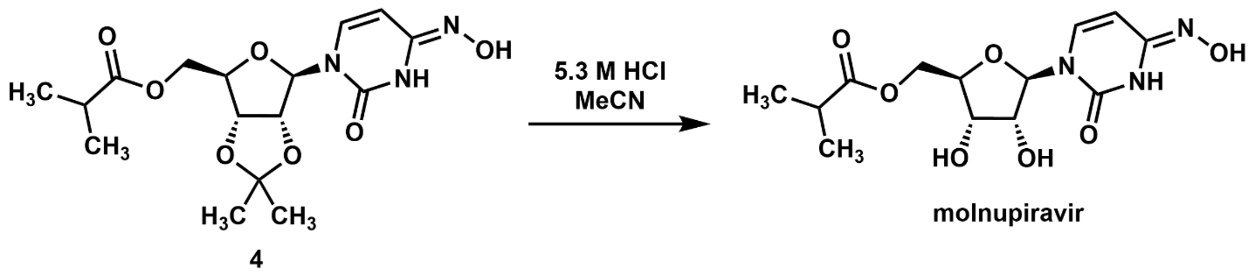

Figure S5: Synthesis of molnupiravir final product from oxime **4**.

To a 5-mL borosilicate glass vial with Teflon-coated stirring bar under ambient atmosphere, was added **Compound 4** (0.171 g, 0.463 mmol, 1.00 equiv) and acetonitrile (1.40 mL). Hydrochloric acid in filtered deionized water (5.3M) was added (75.0 µL, 0.398 mmol, 0.86 equiv) with rapid stirring for a 12-hour reaction. After the reaction period concluded, the reaction contents were transferred to a separation funnel with ethyl acetate rinses for aqueous washing of the organic layer. The ethyl acetate layer (ca. 40 mL) was washed with $NaHCO_3$ saturated aqueous solution (2x 40 mL), water (2x 40 mL), and once with brine (40 mL). The organic layer was shown to contain a single major product using silica TLC. (Using 10% by vol methanol in dichloromethane, molnupiravir has $R_f = 0.30$.) The organic layer was collected and dried with Na_2SO_4, filtered, and volatiles were removed under reduced pressure to leave molnupiravir as white solid in 72.8% yield (0.111 g, 0.337 mmol) that matched ^{1}H and ^{13}C NMR from the literature.[1]

^{1}H NMR (400 MHz, Methanol-*d*4) δ 6.91 (d, *J* = 8.3 Hz, 1H), 5.82 (d, *J* = 4.8 Hz, 1H), 5.61 (d, *J* = 8.2 Hz, 1H), 4.29 (d, *J* = 2.6 Hz, 3H), 4.13 (t, *J* = 4.6 Hz, 1H), 4.08 (d, *J* = 3.9 Hz, 2H), 2.62 (hept, *J* = 7.1 Hz, 1H), 1.18 (d, *J* = 7.0 Hz, 7H). ^{13}C NMR (100 MHz, Methanol-*d*4) δ 178.26, 151.51, 146.14, 131.74, 99.52, 90.36, 82.58, 74.37, 71.50, 64.94, 49.00, 35.15, 19.37, 19.32.

Analytical HPLC

On a Shimadzu Nexera 40 Series HPLC using an SPD-M30A photodiode array detector outfitted with a Shim-pack VP-ODS (5 µm, 4.6x150 mm) column at 40 °C with a 1.0 mL/min flow rate, reactions and standards were monitored and analyzed. Mobile phase A was 20 mM ammonium acetate and Mobile phase B was methanol. The gradient program was as follows: Initial 0% B, 2.00 min, 0% B; 43.00 min, 80% B; 48.00 min, 80% B; 49.00 min, 0% B; 54.00 min, 0%B.

Table S1: Retention times for each synthetic intermediate and molnupiravir final product.

Compound ID	Retention Time (RT, min)
Acetal **1**	18.2
Molnupiravir	23.2
Ester **2**	34.3
Oxime **4**	35.4
Triazole **3**	37.6

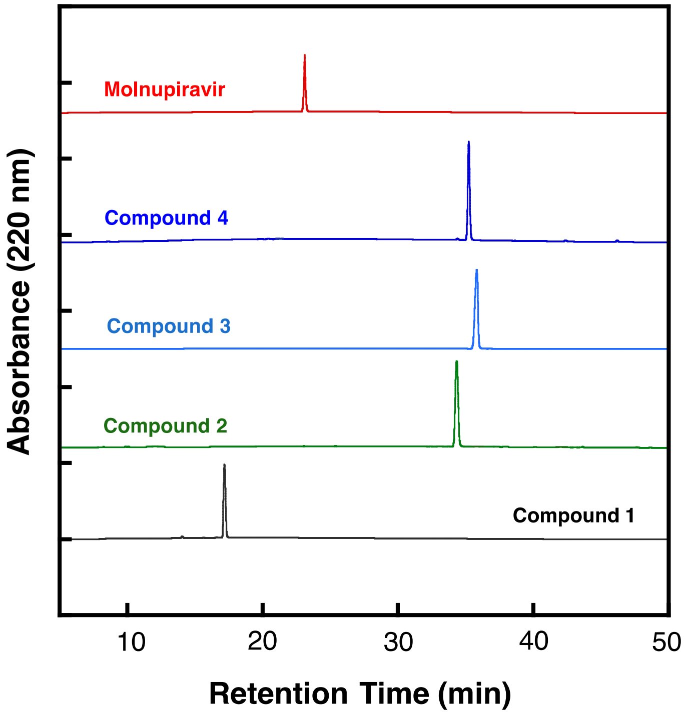

Figure S6: HPLC spectra for synthetic intermediates **1-4** and the molnpiravir final product.

References

1) Fier, P. S.; et. al. The Development of a Robust Manufacturing Route for Molnupiravir, an Antiviral for the Treatment of COVID-19. *Org. Process Res. Dev.* **2021**, *25*, 2806–2815.

^{1}H and ^{13}C NMR Data

(a)

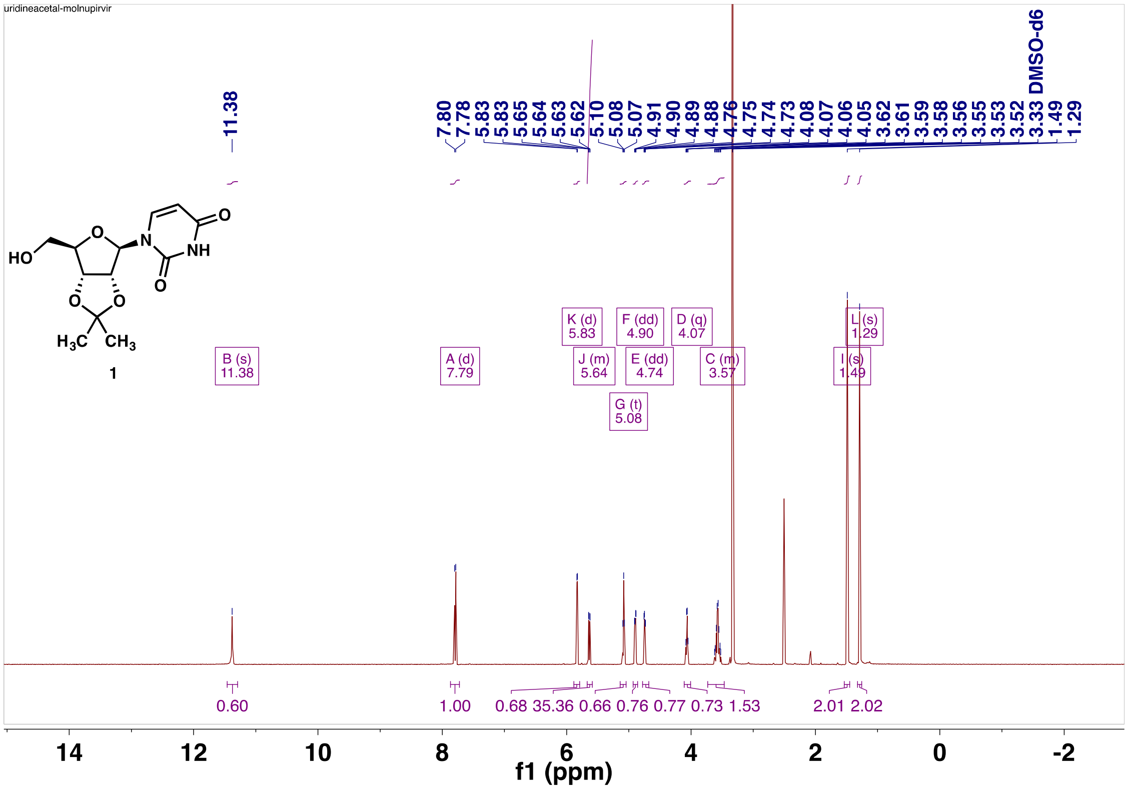

(b)

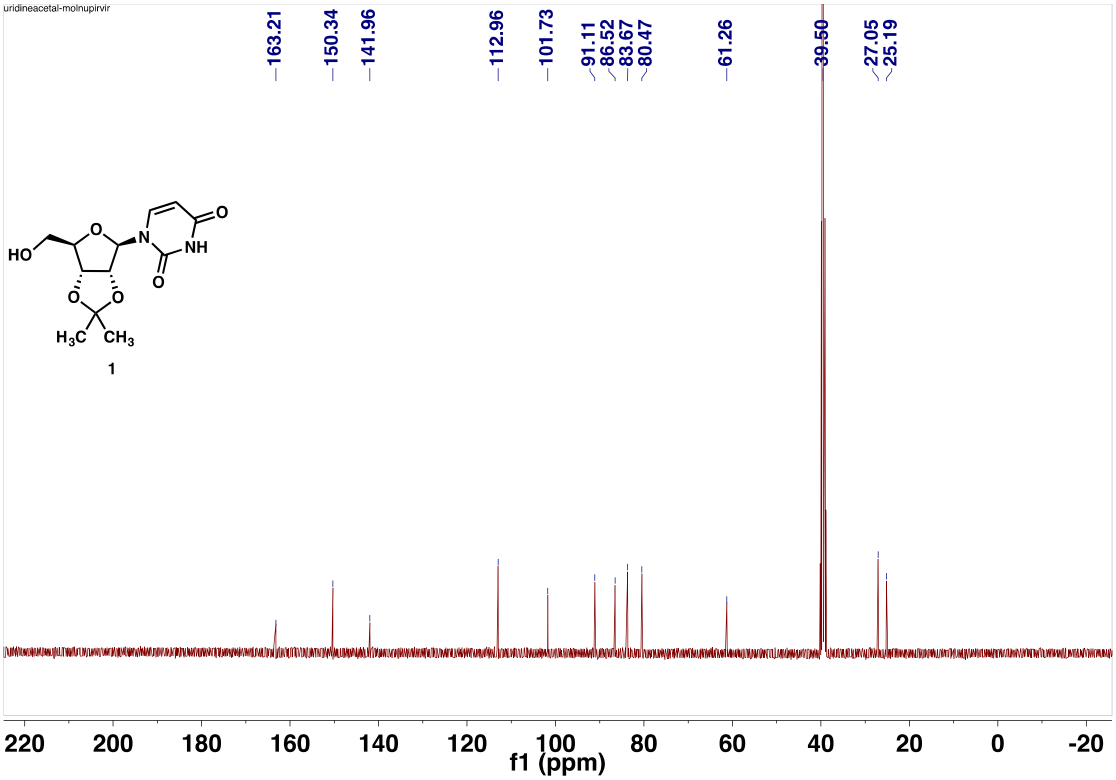

Figure S7: (a) ^{1}H NMR and (b) ^{13}C NMR spectroscopy data of acetal **1**.

(a)

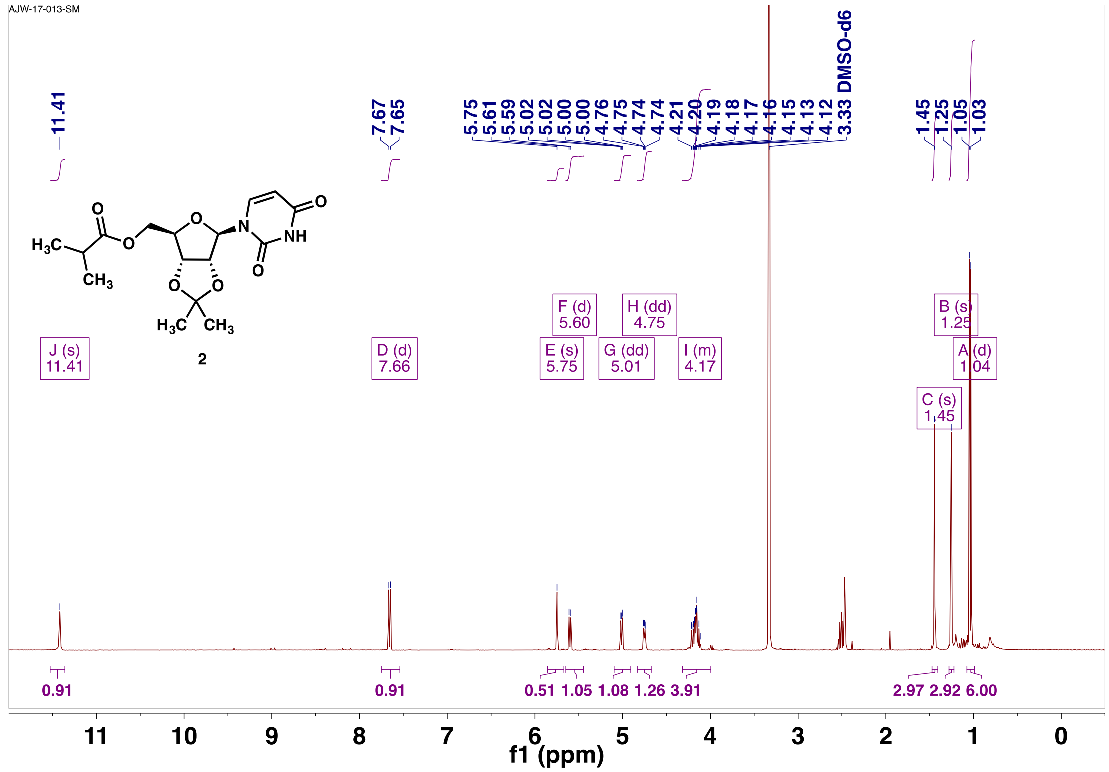

(b)

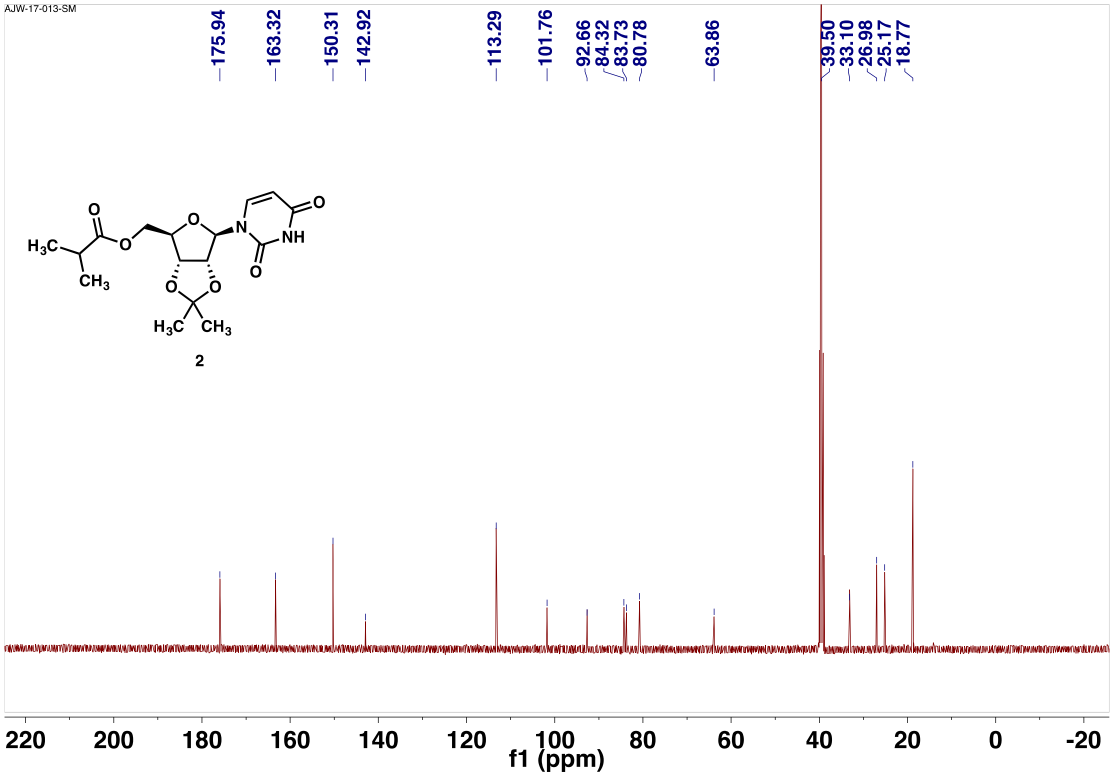

Figure S8: (a) ^{1}H NMR and (b) ^{13}C NMR spectroscopy data of ester **2**.

(a)

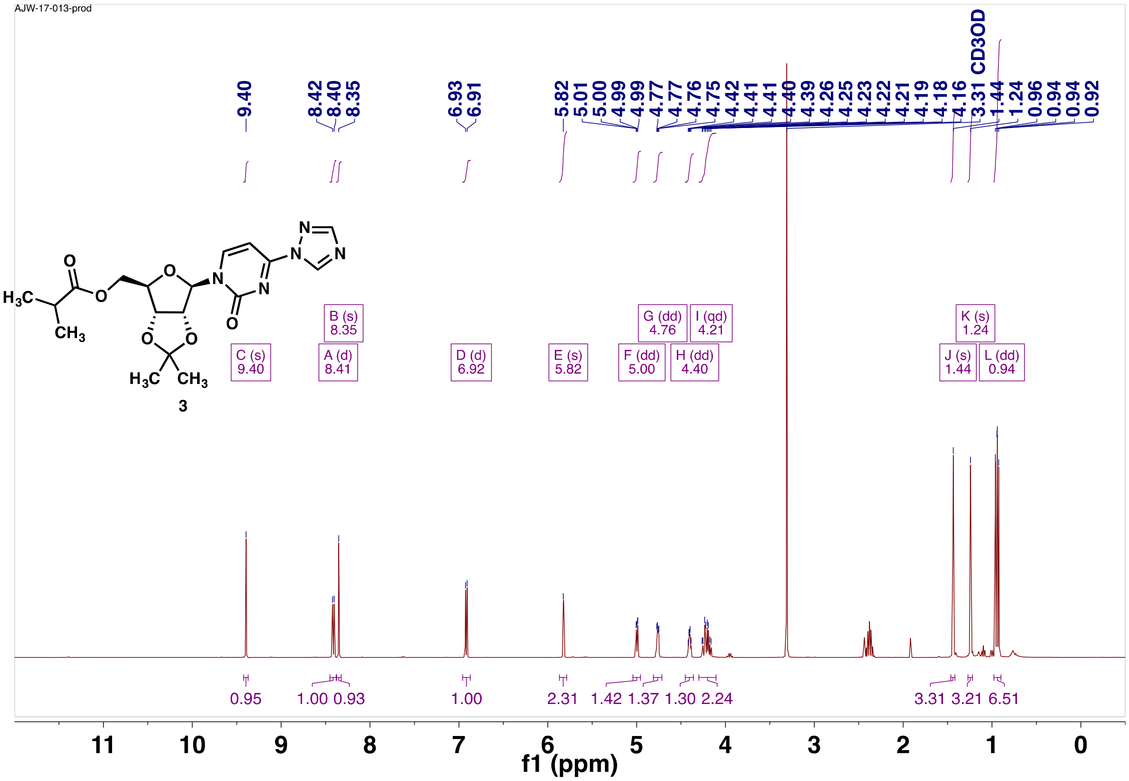

(b)

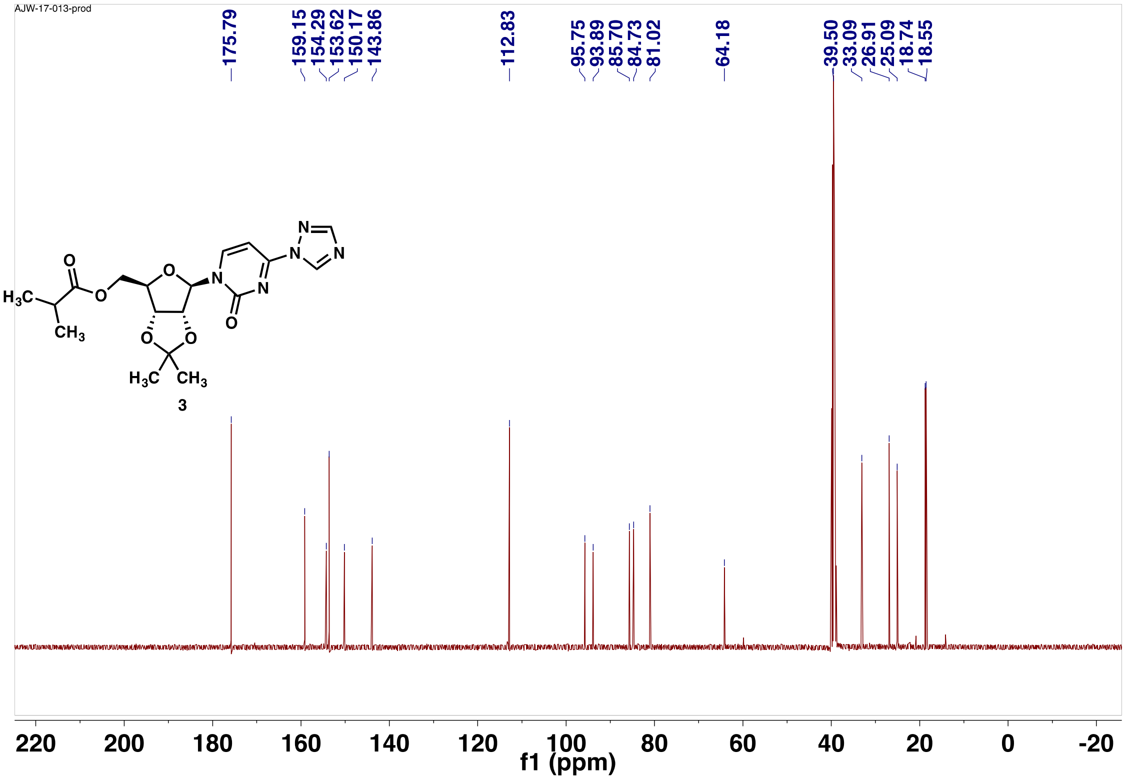

Figure S9: (a) ^{1}H NMR and (b) ^{13}C NMR spectroscopy data of triazole **3**.

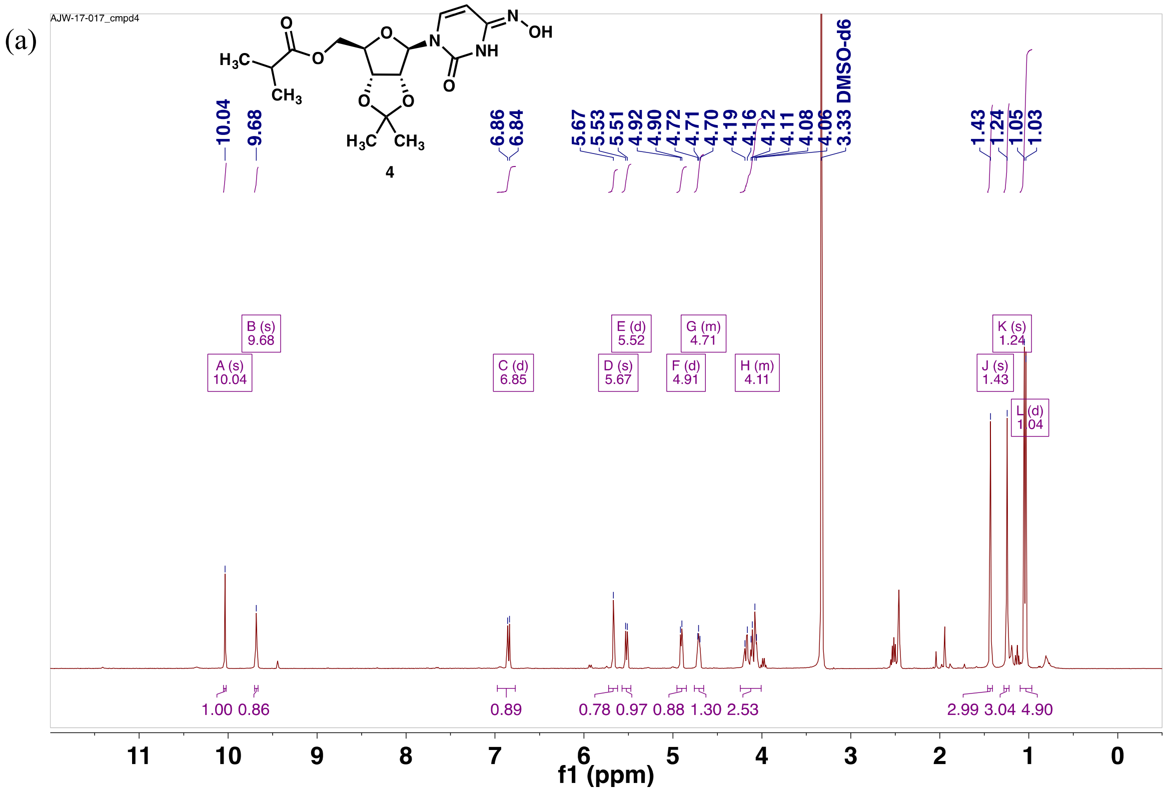

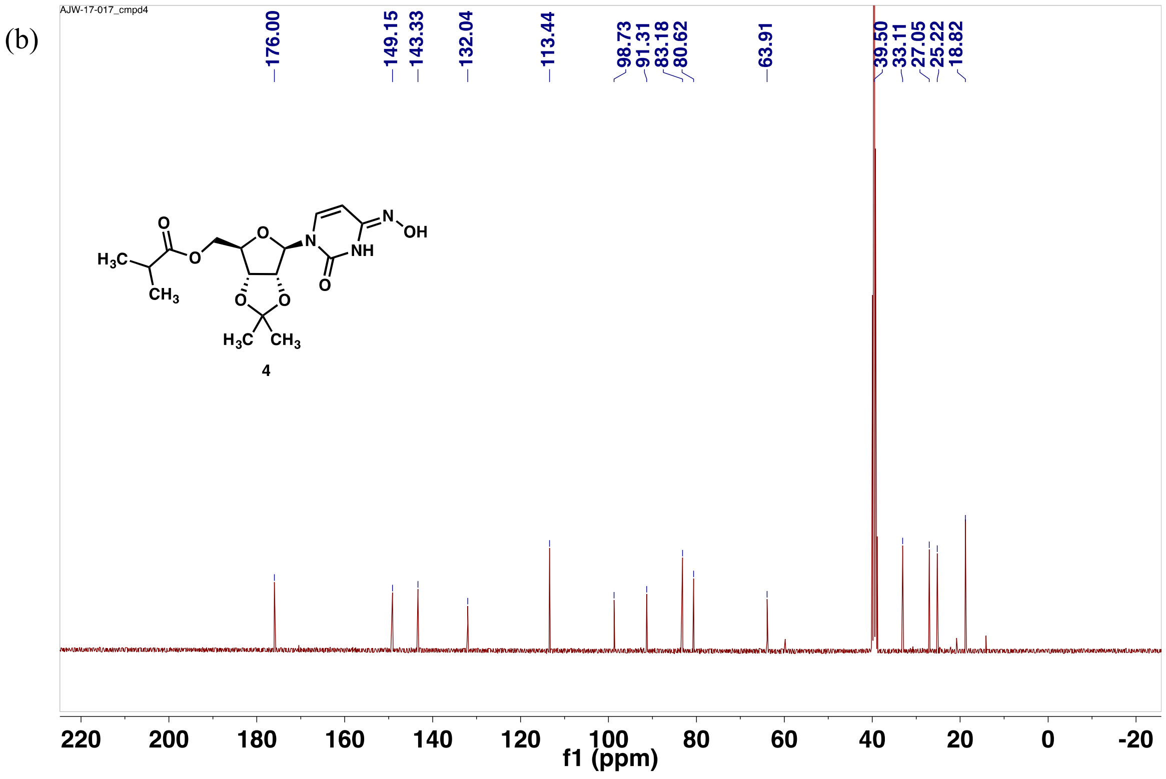

Figure S10: (a) ^{1}H NMR and (b) ^{13}C NMR spectroscopy data of oxime **4**.

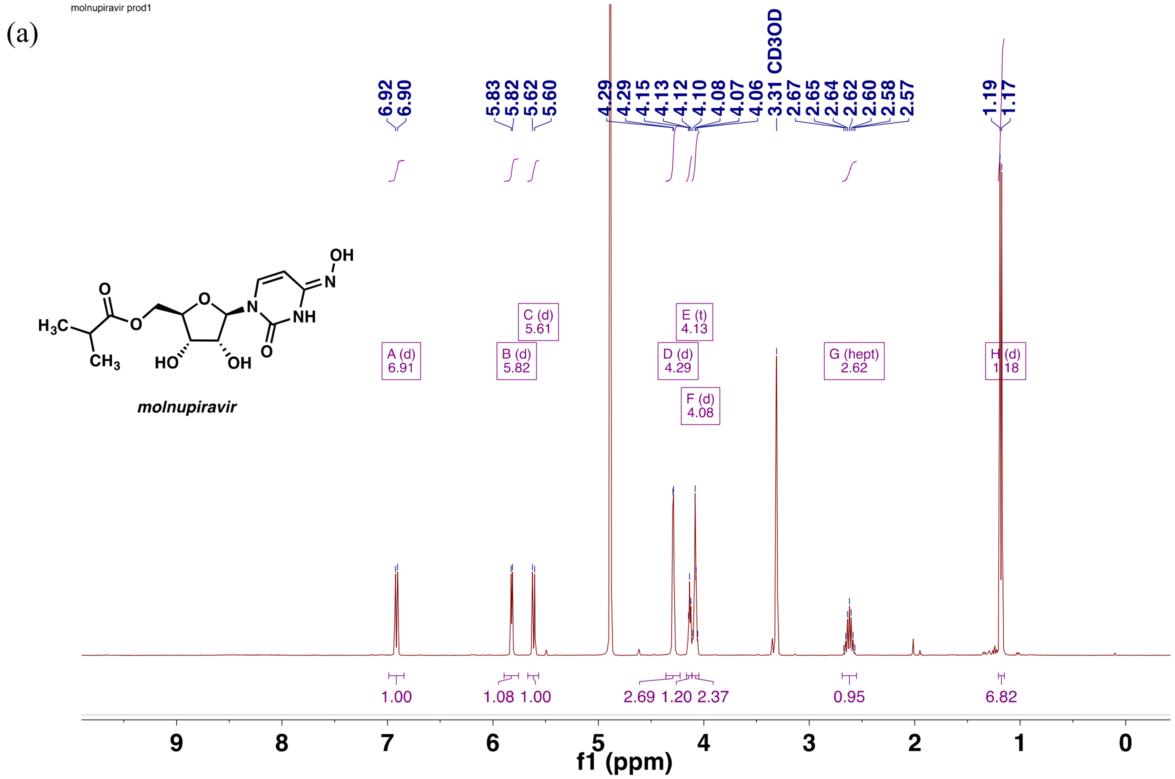

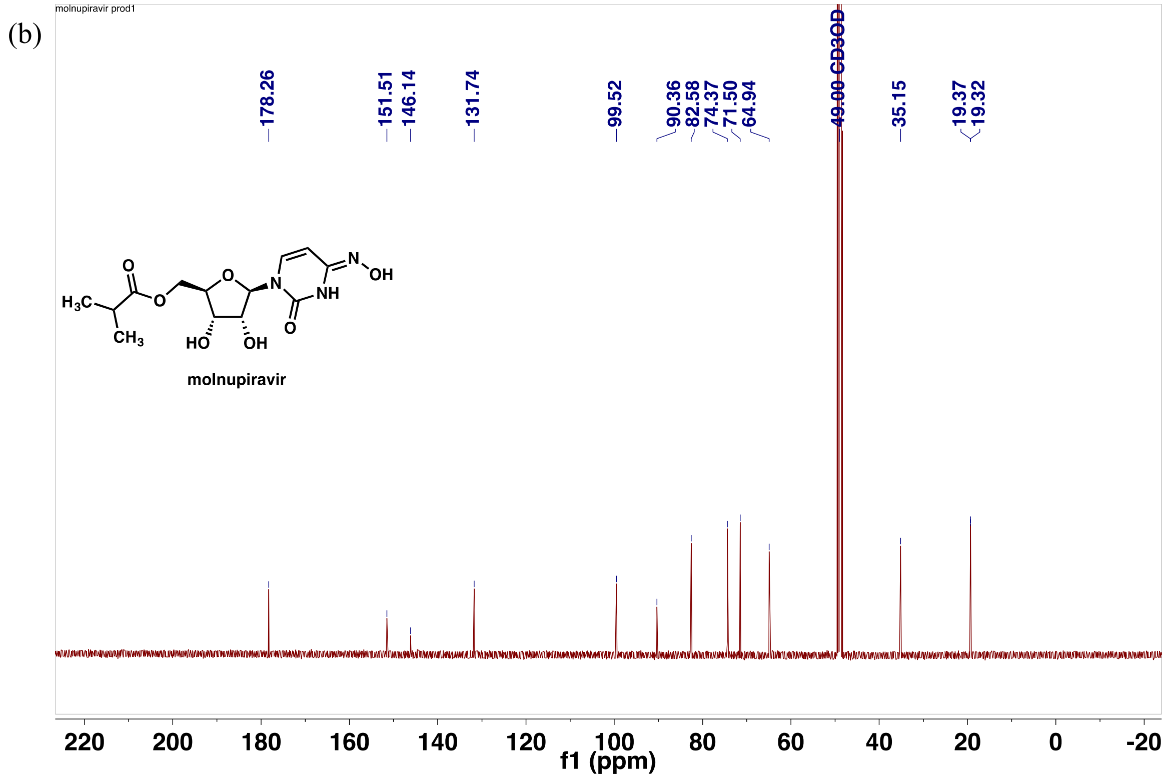

Figure S11: (a) ^{1}H NMR and (b) ^{13}C NMR spectroscopy data of molnupiravir final product.
